# Comparative Analysis of Starch-Based Films Functionalized with *Bixa orellana* Fruit and Leaf Extracts: A Multiscale Structural, Morphological, and Optical Characterization

**DOI:** 10.3390/polym18192341

**Published:** 2026-09-25

**Authors:** Moisés Gallozzo-Cárdenas, Renny Nazario-Naveda, Luis Angelats-Silva

**Affiliations:** 1 Instituto y Centros de Investigación, Universidad Cesar Vallejo, Trujillo 13001, Peru; 2 Departamento de Ciencias, Universidad Tecnológica del Perú, Trujillo 13011, Peru; 3 Laboratorio de Investigación Multidisciplinario, Universidad Privada Antenor Orrego, Trujillo 13008, Peru; langelatss@upao.edu.pe

**Keywords:** *Bixa orellana* extracts, film, morphological characterization, optical properties

## Abstract

This study presents a comparative analysis of starch-based active films functionalized with alcoholic extracts from the fruits (AF) and leaves (AL) of *Bixa orellana* (2.5–10% *w*/*w*), evaluating their structural, morphological, optical, mechanical, barrier, and functional performance. A comprehensive multiscale characterization was performed to analyze the effect of phytochemical composition on structural, morphological, optical, mechanical, barrier, and functional performance. UV-Vis spectroscopy confirmed distinct bioactive profiles: fruit extracts exhibited prominent carotenoid absorption bands (bixin and norbixin, 400–550 nm), whereas leaf extracts displayed characteristic chlorophyll peaks (675 nm) alongside high phenolic/flavonoid content (280–400 nm). This structural differentiation directly influenced material behavior. Opacity measurements at 600 nm demonstrated significant UV-Vis photoprotection, reaching a maximum opacity of 0.435 in 10% AF films. Microscopic analyses by optical microscopy (OM), scanning electron microscopy (SEM), and atomic force microscopy (AFM) revealed that AF integrated smoothly into the starch matrix, reducing surface roughness (RMS = 0.192 μm at 10% AF), whereas AL generated rougher, micro-textured surfaces (RMS = 0.694 μm at 10% AL) due to phase interaction variations. FTIR spectroscopy verified hydrogen bonding and non-covalent molecular interactions between starch chains and functional extract molecules. Mechanically, 10% AF films achieved the highest tensile strength (0.326 ± 0.020 MPa), while 5% AL films provided maximum flexibility (43.49 ± 3.5% elongation at break). Furthermore, AL incorporation significantly improved water resistance, limiting net mass change kinetics to under 50% compared to ~120% in pure starch. Soil-induced mass loss tests showed tunable disintegration kinetics over 28 days, with phenolic-dense formulations exhibiting slower mass loss, although gravimetric measurements cannot distinguish between possible microbial mineralization and physical fragmentation. Antimicrobial agar diffusion assays against *Escherichia coli*, *Staphylococcus aureus*, and *Candida albicans* revealed no clear inhibition zones, indicating that the active hydrophobic compounds remain entrapped within the hydrophilic starch matrix rather than diffusing freely into the agar. Overall, this comparative study highlights the tailored photoprotective, mechanical, and moisture-barrier functionalities achievable using distinct parts of *Bixa orellana*, presenting a promising pathway for circular, eco-friendly active packaging and medical materials.

## 1. Introduction

The escalating accumulation of non-biodegradable synthetic polymers in the environment has become one of the most pressing global challenges of the 21st century. Annual plastic production has surpassed 400 million tons, with a significant proportion consisting of single-use packaging materials that persist in ecosystems for centuries [1,2]. These materials fragment into micro- and nanoplastics, which bioaccumulate across trophic levels, disrupt endocrine functions, and serve as vectors for pathogens and persistent organic pollutants [3,4]. The urgency of this problem has been further highlighted by recent global events; for instance, the COVID-19 pandemic alone generated an estimated 8.4 million tons of additional plastic waste, predominantly from personal protective equipment (PPE) such as masks and gloves [5]. This has, in turn, intensified efforts by the scientific community to develop sustainable, biodegradable alternatives derived from renewable biomass sources, aligning with circular economy principles [6,7]. Despite these advances, the incorporation of plant-derived bioactive compounds into starch films remains a challenge due to the variability in phytochemical composition depending on the plant organ used, which directly influences the final properties of the material.

Among the various biopolymers explored, starch has emerged as one of the most promising candidates for film-forming applications due to its abundance, low cost, disintegration, and excellent filmogenic properties [8,9]. Corn starch, in particular, has been widely investigated for the development of edible and biodegradable films for food packaging and biomedical applications [10]. However, native starch films exhibit inherent limitations, including high hydrophilicity, poor mechanical resistance, low water vapor barrier performance, and lack of functional bioactivity [11,12]. These shortcomings necessitate the incorporation of additives, such as plasticizers (e.g., glycerol) and crosslinking agents, as well as the integration of bioactive compounds to impart active functionality [13,14].

Plant-derived bioactive compounds have gained considerable attention as functional additives for biopolymer films, offering antioxidant, antimicrobial, and photoprotective properties without compromising disintegration [15,16]. Phenolic compounds, flavonoids, carotenoids, and essential oils have been successfully incorporated into starch-based matrices to enhance their performance [17,18]. For instance, starch films containing green tea extract have demonstrated improved antioxidant activity and UV-blocking capacity [19], while those enriched with oregano essential oil have shown significant antibacterial activity against foodborne pathogens [20]. The incorporation of plant extracts not only enhances functional properties but also influences the structural and morphological characteristics of the films, as the molecular interactions between the polymer chains and the bioactive compounds can alter the material’s mechanical behavior, surface roughness, water affinity, and mass loss kinetics [21,22].

Among the vast diversity of plant sources, *Bixa orellana* L. (commonly known as annatto or achiote) stands out as a particularly valuable species native to the tropical regions of the Americas. Its seeds are commercially exploited for the production of bixin and norbixin, natural carotenoid pigments widely used in the food industry as colorants (E160b) [23,24]. Beyond their coloring properties, these carotenoids exhibit strong UV-visible light net mass change kinetics (200–550 nm), making them excellent photoprotective agents for packaging applications [25]. Additionally, *Bixa orellana* accumulates a rich array of secondary metabolites, including tocotrienols, flavonoids, phenolic acids, and terpenoids, which contribute to its antioxidant and antimicrobial activities [26,27]. Studies have reported antifungal activity of *Bixa orellana* seed extracts against *Candida albicans* [28] and antibacterial effects against various Gram-positive and Gram-negative bacteria [29,30]. Furthermore, the plant has a favorable toxicological profile, supporting its safe use in food-contact applications [31,32].

Despite these favorable attributes, most studies on *Bixa orellana* have focused on its seeds and fruits, while the leaves—which are rich in chlorophyll, phenolic compounds, and flavonoids—remain largely unexplored as a source of functional additives for biopolymer films [33,34]. The biochemical composition of plant organs varies significantly: fruits are predominantly rich in carotenoids (bixin and norbixin), whereas leaves contain chlorophyll, higher levels of phenolic compounds, and flavonoids with distinct antioxidant properties [35,36]. This phytochemical divergence suggests that fruit and leaf extracts would interact differently with polymer matrices, potentially yielding films with contrasting structural, morphological, and functional characteristics. The phytochemical divergence between *Bixa orellana* organs is not merely a matter of compositional interest but has direct implications for the structural and functional properties of starch-based films. Carotenoids (bixin and norbixin), which predominate in fruits, are highly hydrophobic, planar molecules that can intercalate between starch chains, potentially reinforcing the polymer network through hydrophobic interactions and π-π stacking with the amylose helix [23,24]. In contrast, the phenolic compounds and flavonoids abundant in leaves possess multiple hydroxyl groups that can form extensive hydrogen bonding networks with starch hydroxyls, likely leading to different molecular arrangements and film morphologies [35,36]. Furthermore, chlorophyll, unique to leaves, contains a porphyrin ring with a central magnesium ion that may coordinate with starch oxygen atoms, introducing distinct optical absorption characteristics [27,28]. These contrasting molecular interaction mechanisms suggest that fruit and leaf extracts would not only impart different colors but also differentially affect film microstructure, mechanical behavior, water affinity, and barrier performance. 

Understanding this structure-function relationship is critically important for the rational design of active packaging materials. From a practical perspective, the choice of plant part determines not only the bioactive profile but also the physical properties of the final material, enabling the tailoring of films for specific applications. For instance, fruit-derived carotenoids may be preferred for UV-protective packaging requiring mechanical strength, while leaf-derived phenolics might be more suitable for moisture-barrier applications. Moreover, the valorization of both fruits and leaves aligns with circular economy principles by maximizing the utilization of agricultural biomass, reducing waste, and providing economically viable alternatives to synthetic packaging [6,7]. Therefore, this systematic comparative study is essential to establish a scientific basis for the informed selection of plant organs in the development of functional biopolymer films.

Advanced characterization techniques, such as Fourier Transform Infrared Spectroscopy (FTIR), Scanning Electron Microscopy (SEM), and Atomic Force Microscopy (AFM), are essential for elucidating the molecular interactions, surface morphology, and structural homogeneity of bio-composite films [37,38]. These techniques enable the validation of successful extract incorporation and provide critical insights into the structure-function relationships that govern the mechanical, barrier, and bioactive performance of the films [23,39].

In this context, the present study was designed to address the gap in the literature regarding the comparative effects of *Bixa orellana* fruit and leaf extracts on the physicochemical and functional properties of starch-based films. We hypothesized that the biochemical differences between fruit (rich in carotenoids such as bixin and norbixin) and leaf (rich in chlorophyll, phenolics, and flavonoids) extracts would result in distinct structural, morphological, optical, and mechanical behaviors when incorporated into a corn starch matrix. Therefore, the primary aim of this work was to systematically investigate the effect of extract type (AF vs. AL) and concentration (2.5, 5.0, 7.5, and 10% *w*/*w*) on: (1) the optical properties (UV-Vis absorbance and opacity); (2) surface morphology and roughness (optical microscopy, SEM, and AFM); (3) molecular interactions between the starch matrix and bioactive compounds (FTIR); (4) mechanical performance (tensile strength and elongation at break); (5) Net mass change kinetics; (6) soil disintegration; and (7) antimicrobial activity against *Escherichia coli*, *Staphylococcus aureus*, and *Candida albicans*. By providing the first comprehensive comparative analysis of *Bixa orellana* fruit and leaf extracts as functional additives in starch-based films, this study aims to establish a sustainable pathway for valorizing agricultural by-products in the development of bioactive packaging materials and biomedical applications.

## 2. Materials and Methods

### 2.1. Preparation of Bixa orellana Extracts

To obtain the extract, the fruit and the leaf of the *Bixa orellana* were ground separately until obtaining powder. The resulting powder was diluted in 96% ethanol (Alkofarma, Lima, Perú) at a ratio of 1:4.5 (*w*:*v*). A 96% ethanol was used because it is a common solvent for the extraction of phenolic compounds and carotenoids. The mixture was subjected to ultrasonication for 30 min and then allowed to stand for 48 h. After this time, it was again subjected to ultrasonication for 30 min and then filtered (Whatman 42/125mm/CAT1442-125, Whatman, Kent, UK). The result obtained was stored at 4 °C for later use.

### 2.2. Manufacture of the Films

To manufacture the films, 2 g of corn starch (Spectrum Chemical Mfg. Corp., New Brunswick, NJ, USA, CAS: 9005-84-9), according to the method described by Nazario et al. [22], was dispersed in 20 mL of distilled water under magnetic stirring with RT Basic Magnetic Stirrer (Thermo Fisher Scientific Inc., Waltham, MA, USA) for 10 min. Subsequently, 1 mL of glycerin (J.T. Baker, Philipsburg, NJ, USA 99%, CAS: 56-81-5) was added dropwise as a plasticizer to reduce the brittleness of the films by decreasing intermolecular forces between starch chains and increasing their flexibility [23]. Additionally, 1 mL of acetic acid (Merck, Darmstadt, Germany, 99%, CAS: 64-19-7) was added dropwise. Among various organic acids available for starch modification, acetic acid was specifically chosen for this formulation due to several key advantages: (i) its mild acidic nature promotes starch chain disentanglement and plasticization without causing excessive depolymerization, which would compromise mechanical integrity [40]; (ii) its high volatility facilitates evaporation during drying, minimizing the risk of residual acidity that could affect film stability or organoleptic properties; and (iii) its compatibility with the hydrophilic starch matrix ensures homogeneous dispersion without phase separation, a common issue with bulkier organic acids such as citric or tartaric acid [41]. The acetic acid also serves to adjust the pH of the mixture, favoring the dispersion of starch chains and creating an environment conducive to the formation of a continuous polymeric network. Furthermore, the inclusion of acetic acid may contribute to the antimicrobial properties of the final films, as short-chain fatty acids are known to disrupt microbial cell membranes through pH-dependent and pH-independent mechanisms [42]. The pH was then adjusted to approximately 5.5 by adding 5 mL of NaOH (0.1 M) (Merck (Rahway, NJ, USA), 98%, CAS: 1310-73-2), monitored with a Hach HQ40D pH meter (Hach, Loveland, CO, USA). The annatto extracts (AF or AL) were incorporated at concentrations of 0%, 2.5%, 5%, 7.5%, and 10% (*w*/*w*, based on starch weight). The mixture was then heated to 60 °C under continuous magnetic stirring at 300 rpm to induce starch gelatinization, a crucial step that promotes the disruption of starch granules and the formation of a continuous polymeric matrix. Once gelatinization was complete (evidenced by a transparent and viscous appearance), the mixtures were homogeneously poured into Petri dishes and dried at 50 °C for 48 h to allow solvent evaporation and film formation. After this time, the films were carefully peeled off and stored under controlled conditions prior to characterization (Table 1).

### 2.3. UV-Vis Spectroscopic Characterization

UV-Vis spectrophotometry (Shimadzu UV-1900, Shimadzu Corporation, Tokyo, Japan), spectrophotometer was employed to characterize the optical properties of both the *Bixa orellana* extracts and the films. This technique was selected to identify the presence of bioactive chromophores, such as carotenoids (bixin and norbixin), chlorophyll, and phenolic compounds, based on their characteristic absorption bands in the UsV-Vis region (200–800 nm). Additionally, this analysis allowed the quantification of film opacity by measuring absorbance at 600 nm, which is a critical parameter for evaluating the photoprotective capacity of the films against UV radiation and visible light.

### 2.4. Optical Microscopy

Morphological observations were performed using an Olympus CX43 optical microscope (Olympus Co., Ltd., Tokyo, Japan) at 40× magnification. This technique was used to obtain a preliminary visual assessment of the surface homogeneity, color distribution, and overall structural integrity of the films. Optical microscopy serves as a rapid screening tool to detect macroscopic defects, phase separation, or incomplete starch gelatinization before proceeding to higher-resolution techniques.

### 2.5. Scanning Electron Microscopy (SEM)

Morphological evaluation was performed on 1 × 1 cm^2^ samples using a scanning electron microscope Tescan Vega 3 LMU (TESCAN, Brno, Czech Republic). The samples were coated with a thin layer of carbon to optimize image quality and examined under an accelerated voltage of 5 kV. SEM was employed to analyze the surface topography and microstructure of the films at high resolution. This technique enables the observation of surface irregularities, porosity, cracks, and the dispersion of extract components within the starch matrix, which are directly related to barrier properties, mechanical performance, and the homogeneity of the polymeric network.

### 2.6. Fourier Transform Infrared Spectroscopy (FTIR)

The functional groups of the films were analyzed by Fourier transform infrared spectroscopy (FTIR) using a Thermo Scientific IS50 spectrophotometer (Thermo Fisher Scientific, Madison, WI, USA). For each sample, spectra were obtained with 20 scans in the frequency range of 4000 to 700 cm^−1^ at a resolution of 4 cm^−1^. FTIR analysis was conducted to identify the chemical interactions between the starch matrix and the bioactive compounds present in the annatto extracts.

### 2.7. Atomic Force Microscopy (AFM)

A Bruker Dimension Edge atomic force microscope (Bruker, Billerica, MA, USA) equipped with Nanodrive V8.02 (Bruker, Billerica, MA, USA) was used, employing a silicon-doped antimony lever tip (RTESP) with a resistivity of 0.01–0.025 Ohm-cm, a spring constant of 40 N/m, and a frequency of 300 kHz in tapping mode. The samples (1 cm^2^) were analyzed in terms of their roughness using the Nanoscope Analysis program v8.02 (Bruker, Billerica, MA, USA). AFM was chosen to provide quantitative and three-dimensional topographical information at the nanoscale. This technique allows the precise measurement of surface roughness parameters (RMS), which are critical for understanding how the incorporation of extracts affects the film’s surface properties, including adhesion, wettability, barrier performance, and the potential release of active compounds.

### 2.8. Mechanical Testing: Tensile Strength and Elongation at Break

Stress and strain tests were carried out according to ASTM D638 [43], using a tensile testing system at a constant speed of 2 mm/s. The applied stress was recorded by a Vernier Dual-Range force sensor (Vernier Software & Technology, Beaverton, OR, USA) with an accuracy of ±0.01 N. The samples were manufactured as V-shaped dumbbell specimens, with a width of 3.18 ± 0.5 mm. Three replicates were performed for each film. Mechanical testing was performed to evaluate the resistance and flexibility of the films, which are essential properties for their practical application in packaging or biomedical materials. Tensile strength indicates the maximum load the film can withstand before failure, while elongation at break reflects its ductility and ability to deform without fracturing. These parameters are directly influenced by the molecular interactions between starch chains and the extract components.

### 2.9. Net Mass Change Kinetics and Component Leaching Profile

Net mass change kinetics were determined by the method described by Nazario et al. [22]. The samples were cut into 2 × 2 cm^2^ pieces and dried at 105 °C for 3 h in an Universal Oven (Memmert GmbH + Co. KG, Schwabach, Germany), and the mass of the dried sample was recorded. The samples were immersed in a beaker with 30 mL of distilled water for 5 s, then the excess water was removed, and the film weight was measured. Three replicates were performed for each film (n = 3), and the results were expressed as mean ± standard deviation (SD). This procedure was repeated until the weight reached a constant value. Water absorption was calculated as the ratio of wet weight to dry weight. This analysis was conducted to evaluate the hydrophilicity of the films, which is a key factor in determining their suitability for packaging applications. The water absorption capacity affects the dimensional stability, barrier properties, and disintegration of the films, and it is directly influenced by the incorporation of hydrophilic or hydrophobic compounds from the extracts.

### 2.10. Film Thickness

The thickness of the films was obtained by averaging five measurements taken at different points, using a digital Vernier Caliper (Truper S.A. de C.V., Mexico City, Mexico) with an accuracy of ±0.01 mm. Thickness measurement is essential because it directly influences other film properties, including optical transparency, mechanical resistance, and water vapor permeability. Thickness also reflects the solid content and the degree of molecular packing after solvent evaporation, which can be affected by the addition of extracts.

### 2.11. Soil-Induced Mass Loss and Disintegration

Soil-induced mass loss and disintegration were determined following the method adapted from Nazario et al. [24]. with modifications. The soil used for the soil burial mass loss test was collected from a cultivated agricultural field located in Trujillo, Peru (coordinates: 8°06′43″ S, 79°03′22″ W), at a depth of 5–15 cm. The soil was characterized prior to use: pH 6.8 ± 0.2 (measured in a 1:2.5 soil:water suspension), organic matter content of 3.5 ± 0.3% (determined by the Walkley–Black method), and a sandy loam texture (68% sand, 22% silt, 10% clay). The soil was sieved through a 2 mm mesh to remove stones and plant debris, and its moisture content was adjusted to approximately 60% of the water-holding capacity by adding distilled water, which was maintained throughout the experiment by periodic watering.

For the disintegration assay, the starch-based films (2 × 2 cm^2^ fragments) were dried at 50 °C for 24 h in Universal Oven (Memmert GmbH + Co. KG, Schwabach, Germany), and the weight (W_t_) was recorded using an PR Analytical Balance (OHAUS Corporation, Parsippany, NJ, USA). The specimens were buried at a depth of 2 cm in plastic containers (20 cm × 15 cm × 10 cm) containing 1.5 kg of the prepared soil. The containers were incubated at 25 ± 2 °C in the dark to simulate natural soil conditions. No external microbial inoculum was added; mass loss was driven by native soil microbiota and abiotic processes; the specific contribution of microbial mineralization vs. abiotic processes was not determined (bacteria, fungi, and actinomycetes) present in the agricultural soil, as described in previous studies.

At intervals of 7, 14, 21, and 28 days, the films were carefully retrieved from the soil, gently rinsed with distilled water to remove soil particles, dried at 50 °C for 24 h, and weighed (W_t_), where Wt represents the dry mass of the film at each specific time interval (t = 7, 14, 21, or 28 days). The percentage of mass loss was calculated using the following equation:

Mass loss (%) = [(W_0_ − W_t_)/W_0_] × 100

where

W_0_ = initial dry mass of the film (at time zero);

W_t_ = dry mass of the film at time t (after 7, 14, 21, or 28 days).

All measurements were performed in triplicate (n = 3), and the results were expressed as mean ± standard deviation (SD). The soil burial mass loss test was conducted over a period of 28 days, as preliminary tests indicated that the control sample (pure starch) reached complete recoverable mass loss within this timeframe.

It is important to note that this gravimetric method measures the loss of recoverable sample mass, which may result from a combination of (i) microbial mineralization (potential enzymatic cleavage of polymer chains by microorganisms), (ii) physical disintegration into small fragments that are not recovered during washing, and (iii) dissolution of water-soluble components. While mass loss in soil is a commonly used indicator of disintegration in starch-based materials [44,45], the absence of complementary measurements such as CO_2_ evolution or residual polymer analysis means that the results should be interpreted as “soil-induced mass loss” or “disintegration” rather than definitive biodegradation/mineralization. This limitation is acknowledged, and future studies should incorporate respirometric methods to confirm true mineralization.

### 2.12. Antimicrobial Activity

To determine the antimicrobial performance of the AF and AL films, the broth microdilution technique was employed, following Clinical and Laboratory Standards Institute (CLSI) protocols. The assay evaluates microbial susceptibility to a concentration gradient of the films in microtiter plates. Reference strains, including *Escherichia coli* (ATCC 25922), *Staphylococcus aureus* (ATCC 29213), and *Candida albicans* (ATCC 10231), were incubated for 18 to 24 h. Microbial cultures were then suspended in sterile physiological saline to match the 0.5 McFarland turbidity standard (1 × 10^8^ CFU/mL). Following serial dilution, 10 μL of inoculum was transferred into each well, yielding a final working concentration of 1 × 10^5^ CFU/mL. Microplates were incubated in IN30 Incubator (Memmert GmbH + Co. KG, Schwabach, Germany) at 36 ± 1 °C for 24 to 48 h before evaluating microbial growth. Tests involving bacterial strains (*E. coli* and *S. aureus*) were performed in triplicate, while fungal assays (*C. albicans*) were carried out in duplicate [25]. Antimicrobial testing was conducted to evaluate the potential of *Bixa orellana* extracts as bioactive agents that can inhibit the growth of pathogenic microorganisms. This property is of great interest for the development of active food packaging and biomedical materials, as it can extend the shelf life of products and prevent infections. The selection of *E. coli*, *S. aureus*, and *C. albicans* represents common foodborne and clinically relevant pathogens.

## 3. Results

### 3.1. UV-Vis Spectroscopic Analysis of Extracts

Figure 1 shows the UV-Vis absorbance spectra of the alcoholic extracts from *Bixa orellana* fruits (AF) and leaves (AL). These spectra provide preliminary insights into the chromophore composition of each extract; however, it is important to acknowledge that UV-Vis spectroscopy of crude plant extracts typically yields broad, overlapping absorption bands due to the presence of multiple absorbing species. Therefore, the following band assignments are based on literature precedents and should be interpreted with caution, as definitive compound identification would require complementary chromatographic techniques.

In the 400–550 nm range, both extracts exhibit notable absorbance. The AF extract shows two distinct shoulders in this region, which is consistent with the presence of carotenoids—specifically bixin and norbixin, the main pigments of *Bixa orellana* fruits [26]. However, other metabolites such as phenolic compounds may also contribute to absorbance in this spectral window, and the observed bands cannot be exclusively attributed to carotenoids based solely on these data. The AL extract also displays absorbance in this range, although with a lower intensity compared to AF, suggesting a relatively lower carotenoid content in leaves.

A strong and broad absorbance band is observed between 280 and 400 nm for both extracts, which is commonly attributed to phenolic compounds and flavonoids [29,30,31]. The AL extract exhibits comparatively higher absorbance in this UV region than the AF extract, which may indicate a richer phenolic/flavonoid profile in the leaves, although quantitative differences would need to be confirmed by specific colorimetric assays (e.g., Folin–Ciocalteu for total phenolics or aluminum chloride for flavonoids).

In the leaf extract spectrum, a small but discernible peak is observed around 675 nm. This feature is consistent with the characteristic absorption of chlorophyll [27,28] and is absent in the AF spectrum, as expected given the distinct physiological roles of these organs. Nevertheless, given the complexity of the crude extract, this band could potentially overlap with other minor metabolites, and definitive confirmation of chlorophyll would require further analytical separation.

Overall, the spectral differences between AF and AL—particularly the stronger visible-region absorbance of AF (400–550 nm) and the stronger UV-region absorbance and 675 nm feature of AL—reflect the distinct phytochemical profiles of the fruit and leaf. The pronounced absorbance in the UV-B/UV-A range (280–400 nm) for both extracts suggests their potential to act as photoprotective agents when incorporated into starch-based films, as they could attenuate radiation that promotes oxidative disintegration of packaged products [32,33]. However, the actual photoprotective performance in the final films depends on the molecular interactions with the polymer matrix, which are further explored in the following sections through FTIR and opacity measurements.

### 3.2. Film Opacity

Figure 2 presents the UV–Vis absorbance spectrum of corn starch-based films containing different concentrations of AF and AL. The analysis was conducted in the 550–650 nm range to determine absorbance at 600 nm, which was then used to calculate the opacity of the films. The figure shows a progressive increase in absorbance with higher extract concentrations, indicating that the films become more opaque as more extract is incorporated. According to the literature, this effect can be attributed to the presence of phenolic compounds in AF and AL, particularly bixin, since extracts with higher bixin content tend to exhibit more intense coloration, thereby influencing opacity [32,34,35]. 

The 10% AF sample recorded the highest opacity value (0.435 ± 0.012 at 600 nm), which was significantly greater than the control and all other formulations (*p* < 0.05, ANOVA with Tukey post hoc test). Similarly, the 10% AL sample displayed relatively high absorbance compared to other AL films; however, the AF film samples consistently showed higher absorbance values, which can be explained by their greater concentration of secondary metabolites with active chromophores. The increase in opacity suggests the physical integration of *Bixa orellana* bioactive components into the starch matrix, resulting in a denser and more heterogeneous structure that hinders photon transmission through the film [35,36,37,38]. 

Opacity is a key property in the development of medical patches and packaging materials, as it functions as a barrier against UV radiation and visible light [23].

### 3.3. Optical Microscopy Image

Figure 3 shows optical micrographs of corn starch-based films (0%) containing 2.5% AF, 5% AF, 7.5% AF, and 10% AF, as well as 2.5% AL, 5% AL, 7.5% AL, 10% AL. The control sample exhibits a uniform and continuous surface, with no defined granular structures. According to previous studies, this suggests complete starch gelatinization, resulting in a continuous amorphous phase film with homogeneous morphology, which may influence its mechanical properties [23]. 

In the AF films at different concentrations, a progressive change in both coloration and microstructure is observed, with greater surface roughness at lower concentrations. Furthermore, increasing extract content leads to higher heterogeneity, which may indicate the incorporation of bioactive compounds. This observation correlates with the spectral profile of the AL, which contains chlorophyll (peak at 670 nm), conferring distinct optical and chemical properties compared to the fruit extract. In AL films, a greater number of irregularities are evident, particularly in the 7.5% and 10% samples, likely due to interactions between the polymeric matrix and the incorporated extract [32,35].

Overall, visual analysis reveals that the films maintain a generally homogeneous appearance, confirming good integration of the components, along with a noticeable change in coloration depending on the type of extract and its concentration. The incorporation of the extracts is further evidenced by a yellowish tone in the AF films and a whitish tone in those containing AL.

### 3.4. Scanning Electron Microscopy (SEM)

Figure 4 shows morphological analysis of the samples: corn starch (0%), 2.5% AF, 5% AF, 7.5% AF, 10% AF and 2.5% AL, 5% AL, 7.5% AL, 10% AL. The starch-only film (control sample) exhibits a smooth surface with minimal irregularities or pores (Figure 4, 0%). In contrast, the surfaces of starch films containing AL appear more irregular and rougher (Figure 4, AL), particularly in the 7.5% and 10% samples. This suggests possible interactions between the polymeric matrix and the phenolic and flavonoid compounds of the leaf, as corroborated by the UV–Vis analysis (see Figure 1).

AF films, on the other hand, display smoother and more uniform surfaces (Figure 4, AF). No agglomerations, cracks, or phase separations are observed in any of the films. The interaction between the polymeric matrix and the carotenoids present in the fruit extract can be inferred, consistent with the UV–Vis analysis (Figure 1).

Overall, it can be concluded that the incorporation of *Bixa orellana* extracts modifies the polymer surface, leading to changes in barrier properties and opacity—previously analyzed—as well as in adhesion, water absorption capacity, elasticity, and interactions with bioactive compounds, which will be further confirmed in subsequent analyses.

### 3.5. Fourier Transform Infrared Spectroscopy (FTIR)

The FTIR spectra of the films are shown in Figure 5 and Figure 6, providing insights into the molecular interactions between the starch matrix and the *Bixa orellana* extracts. All spectra exhibited characteristic absorption bands of starch-based materials, with subtle changes in intensity and position upon extract incorporation.

O–H stretching region (~3400–3300 cm^−1^): A broad and intense band was observed in all samples, centered around 3400–3300 cm^−1^, which corresponds to the stretching vibrations of hydroxyl (O–H) groups. This band arises from the extensive hydrogen bonding network in starch, involving both intra- and intermolecular O–H interactions between starch chains, as well as bound water molecules within the polymer matrix. Upon incorporation of the extracts, subtle shifts in this band were observed, suggesting the formation of new hydrogen bonds between the hydroxyl groups of starch and the phenolic –OH groups present in the extracts.

C–H stretching region (~2920 cm^−1^): A band near 2920 cm^−1^ was observed, attributed to the asymmetric C–H stretching vibrations of the –CH_2_ groups in the starch backbone. This band is characteristic of the aliphatic structure of polysaccharides and remained largely unchanged upon extract addition, indicating that the starch skeleton was not significantly modified.

Region ~1640 cm^−1^: A band was observed near 1640 cm^−1^ in all spectra. In starch-based films, this band is predominantly assigned to H–O–H bending of water molecules bound within the hydrophilic matrix. Contributions from C=O and C=C stretching of phenolic/flavonoid compounds cannot be excluded, but they are likely minor and overlapped. Therefore, this band should not be used as standalone evidence of C=O/C=C interactions [46]. The subtle changes in this region with extract concentration are consistent with changes in hydration and hydrogen-bonding environment.

C-O stretching region (~1000 cm^−1^): A strong absorption band centered around 1000 cm^−1^ was observed, characteristic of C–O stretching vibrations of the glycosidic bonds (C–O–C) and C–O–H groups in the starch polysaccharide backbone. This band is typical of the polysaccharide skeleton and is sensitive to changes in the local molecular environment. The subtle modifications in this region upon extract addition may reflect alterations in the starch chain conformation and intermolecular hydrogen bonding.

Overall, the FTIR spectra confirm the successful incorporation of *Bixa orellana* extracts into the starch matrix. The observed spectral changes—hydration/hydrogen-bonding region—are consistent with the establishment of non-covalent interactions, primarily hydrogen bonding, between the starch chains and the bioactive compounds present in the extracts. These molecular interactions are responsible for the modifications in the physicochemical properties of the films, including mechanical performance, water resistance, and opacity, as discussed in the following sections [39,47].

### 3.6. Atomic Force Microscopy (AFM)

Figure 7 presents atomic force microscopy (AFM) images of the film morphology, while Table 2 reports the measured values of surface roughness (RMS) for starch-based film samples: 0% (control), and 2.5% AF, 5% AF, 7.5% AF, 10% AF and 2.5% AL, 5% AL, 7.5% AL, 10% AL. The control sample (0%) shows an RMS value of 0.472 µm, indicating a relatively smooth and homogeneous surface with minimal irregularities or marked perturbations.

In the AF films, an increase in roughness is observed, attributed to the formation of small protrusions or an increase in surface porosity. This effect is evident in the 2.5% AF, 5% AF, and 7.5% AF samples. However, in the 10% AF sample, a drastic reduction in roughness is detected, likely due to a smoothing effect at higher extract concentrations, suggesting possible molecular reorganization.

For the AL films, the 5% AL and 10% AL samples exhibit RMS values of 0.624 µm and 0.694 µm, respectively, making them the roughest among the studied films. AFM images corroborate these findings, showing irregular structures caused by cracks and agglomerations. This evidence indicates that *Bixa orellana* leaf extract does not integrate effectively into the polymeric matrix.

When comparing the incorporation of both extracts, it was observed that *Bixa orellana* fruit extract integrates into the polymeric matrix up to 10%. Beyond this concentration, surface roughness increases noticeably. In contrast, films containing *Bixa orellana* leaf extract exhibit a faster rise in roughness, indicating greater alterations to the surface.

These differences are functionally significant: lower roughness is associated with higher resistance to moisture and greater transparency, whereas increased roughness enhances coating adhesion and may facilitate the release of active compounds.

### 3.7. Mechanical Properties: Tensile Strength and Elongation at Break

Figure 8 and Figure 9 present the stress–strain curves and the corresponding tensile properties of the starch-based films. The numerical results are summarized in Table 3. A detailed examination of the data reveals a strongly non-monotonic mechanical response to the incorporation of both AF and AL extracts, rather than a simple concentration-dependent reinforcement or softening.

For AF films, the tensile strength exhibited a fluctuating trend: it decreased from the control value of 0.205 ± 0.015 MPa to 0.115 ± 0.010 MPa at 2.5% AF, partially recovered to 0.181 ± 0.012 MPa at 5% AF, dropped sharply to a minimum of 0.096 ± 0.008 MPa at 7.5% AF, and finally reached its maximum value of 0.326 ± 0.020 MPa at 10% AF. Similarly, the elongation at break for AF films showed an initial increase from 31.36 ± 2.1% (control) to 39.54 ± 3.0% at 5% AF, followed by a marked decrease to 20.54 ± 1.9% at 7.5% AF, before recovering to 34.57 ± 2.5% at 10% AF.

A comparable non-linear behavior was observed for AL films. The tensile strength declined to 0.115 ± 0.009 MPa at 2.5% AL, improved to 0.192 ± 0.014 MPa at 5% AL, and then plateaued at lower values of approximately 0.135 MPa for 7.5% and 10% AL. The elongation at break for AL films reached its highest value at 5% AL (43.49 ± 3.5%), but decreased considerably to 26.53 ± 2.2% and 24.49 ± 2.0% at 7.5% and 10% AL, respectively.

Overall, these non-linear trends indicate that the mechanical properties of the *Bixa orellana*-functionalized films are governed by a complex interplay of plasticization, molecular crosslinking, and phase heterogeneity, which vary with extract type and concentration. These mechanisms are further discussed in Section 4.7.

Table 3, Figure 8 and Figure 9 evaluate the mechanical performance of films of corn starch (0%), 2.5% AF, 5% AF, 7.5% AF, 10% AF and 2.5% AL, 5% AL, 7.5% AL, 10% AL, revealing how these natural additives can be tailored to adjust the mechanical properties of the material toward greater strength or flexibility. Figure 7 presents the stress–strain curves. The 10% AF sample exhibited the highest tensile strength, while the 5% AL sample showed the greatest elongation. This trend is further detailed in the extract-specific figures: Figure 9 shows that elongation at break reaches its maximum precisely at 5% AL, maintaining a tensile strength comparable to the control but decreasing sharply at higher concentrations (7.5% and 10%), suggesting that excessive extract disrupts the cohesion of the polymeric matrix. Conversely, Figure 8 illustrates a divergent behavior, with tensile strength progressively increasing and peaking at 10% AF, significantly surpassing all other samples. This demonstrates that fruit-derived components act as effective reinforcing agents at this specific dosage.

Numerical results summarized in Table 3 confirm these observations: the control sample exhibits a tensile strength of approximately 0.205 MPa and an elongation of about 31.35%. The 10% AF sample is the strongest material in the study (≈0.326 MPa), while the 5% AL sample is the most flexible (elongation ≈ 43.49%). The table also highlights the overall deterioration of mechanical properties in the 10% AL sample (≈0.135 MPa tensile strength and ≈24.49% elongation).

In conclusion, the incorporation of *Bixa orellana* extracts not only modifies starch properties but also enables targeted adjustments: 10% AF is the optimal formulation for applications requiring higher mechanical strength to withstand loads, whereas 5% AL is ideal for applications demanding greater elasticity and adaptability without fracture.

### 3.8. Net Mass Change Kinetics and Component Leaching Profile

Figure 10A,B present the evolution of the net mass change in the starch-based films upon immersion in distilled water over time. It is critical to interpret these data with caution: the measured mass change represents the net balance between two simultaneous and competing processes: (i) water absorption (mass gain) into the hydrophilic polymer matrix, and (ii) dissolution and leaching of water-soluble components (mass loss) from the film, including the plasticizer (glycerol), low-molecular-weight extract constituents (phenolics, flavonoids, and residual sugars), and potentially soluble starch fractions. Therefore, the observed profiles reflect coupled absorption–desorption phenomena rather than purely water uptake kinetics.

All films exhibited an initial rapid increase in net mass, reaching a maximum within the first 20–40 min of immersion. The pure starch film (control) reached the highest peak value, approximately 120 ± 5.2%, consistent with its highly hydrophilic and porous structure. However, following this peak, several formulations—particularly the control and the AF films—showed a progressive and substantial decrease in net mass, indicating that the rate of material dissolution into the aqueous medium began to exceed the rate of water absorption.

The AF films exhibited net mass values between 60% and 80% at their peak, but also showed a notable decline after the maximum, suggesting significant leaching of glycerol and/or soluble extract components. In contrast, the AL films displayed the most distinct behavior: all AL-containing formulations maintained net mass values below 50% throughout the immersion period, with a much less pronounced decrease after the initial peak. This suggests that the leaf extract not only limited water uptake—likely through the formation of a denser hydrogen-bonded network—but also reduced the leaching of soluble components by effectively entrapping them within the reinforced polymer matrix.

Overall, these results demonstrate that the incorporation of *Bixa orellana* extracts—particularly the leaf extract—substantially reduces both water uptake and the subsequent loss of film components upon exposure to aqueous environments, indicating improved structural integrity and resistance to dissolution.

### 3.9. Film Thickness and Density

Table 4 compares the physical properties of corn starch films. The thickness analysis reveals that the addition of *Bixa orellana* extracts modifies the polymeric matrix in distinct ways. While the pure starch control sample exhibits a baseline thickness of 0.17 mm, the AF films produce the most pronounced variation, reaching a maximum of 0.21 mm at 2.5% concentration, followed by a sharp reduction to 0.10 mm at 5%. This suggests a reorganization of polymer chains at this dosage, with stabilization at 0.18 mm at higher concentrations (7.5% and 10% AF). In contrast, the AL films shows a more consistent integration, with thickness values ranging from 0.16 mm (7.5% AL) to 0.20 mm (5% AL), and returning to 0.17 mm at 10% AL—identical to the control. This indicates that the leaf extract incorporates into the matrix without significantly increasing its volume. According to the literature, thickness is a critical variable as it directly influences functional properties such as opacity (light barrier) and water vapor permeability. Greater thickness generally correlates with enhanced barrier performance, while also reflecting changes in solid content after solvent evaporation. In conclusion, the addition of *Bixa orellana* extracts does not produce a linear effect: fruit extract induces more pronounced variations, likely due to differences in miscibility, whereas leaf extract yields films with dimensions closer to those of the original starch matrix [32,35,48].

### 3.10. Soil-Induced Mass Loss and Disintegration

Figure 11 presents the soil-induced mass loss (disintegration) of pure corn starch films (0%) and films incorporated with AF and AL extracts over 28 days of soil burial. The percentage of mass loss was calculated using the equation detailed in Section 2.11 at each sampling time (7, 14, 21, and 28 days). It is important to emphasize that this gravimetric method measures the loss of recoverable sample mass, which may result from a combination of (i) true mineralization/biodegradation (enzymatic cleavage of polymer chains and mineralization), (ii) physical fragmentation into small pieces that are not recovered during washing, and (iii) dissolution of water-soluble components such as glycerol and low-molecular-weight extract residues. Therefore, the results are interpreted as soil-induced mass loss or disintegration, not as definitive biodegradation/mineralization. The specific contribution of microbial mineralization was not determined in this study, and complementary respirometric measurements (e.g., CO_2_ evolution) would be required to confirm true mineralization/biodegradation.

The mass loss profiles revealed distinct behaviors depending on the extract type and concentration. During the initial phase (days 0–7), the control film (0% starch) exhibited minimal mass loss (1.27 ± 0.3%), reflecting an adaptation period for the soil microbiota and abiotic processes to begin affecting the polymer matrix. In sharp contrast, the film containing 10% AF showed accelerated mass loss from the outset, reaching 23.65 ± 1.8% within the first week. This suggests that, at high concentrations, the fruit extract may act as a readily accessible carbon source that stimulates early microbial or chemical disintegration processes. However, this interpretation remains hypothetical, as the gravimetric method cannot distinguish between biological and abiotic mechanisms.

The time required to reach complete recoverable mass loss varied significantly among formulations. Both the control film and the 2.5% AF formulation reached complete recoverable mass loss within 21 days, demonstrating the inherent susceptibility of starch to soil-induced disintegration. This behavior is consistent with the hydrophilic nature of starch, which can swell and facilitate enzymatic access, but again, the exact contribution of enzymatic hydrolysis versus physical fragmentation and dissolution cannot be resolved from mass loss data alone. The incorporation of higher extract concentrations, particularly leaf extract, introduced a fundamental change in the mass loss kinetics. The AL films exhibited slower and more gradual mass loss over the 28-day period, with no formulation reaching complete recoverable mass loss by the end of the test. This deceleration is likely due to the formation of a denser, more hydrogen-bonded network between the phenolic compounds of the leaf extract and the starch matrix, which may limit both enzymatic access and physical fragmentation. However, this mechanism is proposed as a hypothesis, since the gravimetric method does not provide direct evidence of crosslinking or enzymatic resistance.

The differences in mass loss behavior among the formulations can be summarized as follows:

- Control (0%) and 2.5% AF: rapid mass loss, complete within 21 days, indicating low structural resistance to soil-induced disintegration.
- AF of 5% and 7.5%: intermediate mass loss rates, with slower progression than the control but faster than AL films.
- AF of 10%: accelerated early mass loss (23.65% in 7 days), followed by a more gradual decline, suggesting a dual effect of the fruit extract: initial stimulation of disintegration processes at high concentration, followed by some structural reinforcement.
- AL films (2.5–10%): consistently slower mass loss, with the highest concentrations (7.5% and 10% AL) showing the lowest mass loss rates. This is consistent with a more compact and less accessible polymer network.

These results should be interpreted with caution. The soil used in this study (pH 6.8 ± 0.2, organic matter 3.5 ± 0.3%, sandy loam texture) is representative of agricultural soils in the Peruvian region, and the native microbial community (bacteria, fungi, and actinomycetes) drove the mass loss process without external inoculation. However, the absence of CO_2_ evolution measurements means that the terms “disintegration” and “biodegradability” are avoided throughout this section. The observed trends provide useful comparative information on the relative stability of the films under soil burial conditions, but they do not constitute proof of microbial mineralization.

From a practical standpoint, the tunable mass loss behavior offers opportunities for designing films with controlled lifespans. AF-rich formulations may be suitable for short-term applications where rapid disintegration is desired (e.g., single-use food wrappers), while AL-rich formulations could be used in applications requiring longer functional stability (e.g., agricultural mulching films or biomedical devices that require gradual resorption). Nevertheless, future studies should incorporate respirometric methods, molecular weight analysis of residual polymer, and microscopic examination of soil-incubated samples to confirm true mineralization/biodegradation and distinguish it from physical fragmentation and dissolution.

### 3.11. Antimicrobial Activity

The antimicrobial and antifungal performance of the starch-based films functionalized with AF and AL extracts was evaluated against *Candida albicans*, *Escherichia coli*, and *Staphylococcus aureus* using the agar disc-diffusion technique, as illustrated in Figure 12. The control film (0%) showed no inhibitory effect, allowing normal microbial growth. Overall, the absence of clear, extended inhibition zones around the incorporation sites for both AF and AL formulations suggests a limited migration of the active phytochemicals—such as bixin, norbixin, and polyphenols—from the hydrophilic starch network into the surrounding agar medium, which is characteristic of hydrophobic bioactive compounds bound within a polymer matrix. Despite this restricted diffusion, a contact-inhibitory effect and distinct visual alterations in film opacity and color were observed at higher extract concentrations (7.5% and 10%), particularly against *C. albicans* and *S. aureus*. The higher susceptibility observed in *S. aureus* compared to *E. coli* aligns with the structural differences in their cell walls, as Gram-negative bacteria possess an outer lipopolysaccharide membrane that acts as a barrier against hydrophobic plant extracts.

## 4. Discussion

The performance of the starch-based films functionalized with *Bixa orellana* extracts was evaluated across three distinct complex environments: plant tissue (as a model for food packaging), soil (as a model for disintegration), and culture media (as a model for antimicrobial activity). The results demonstrate that the stability and effectiveness of the films are highly dependent on both the type of extract (AF vs. AL) and the environmental conditions.

### 4.1. Stability and Effectiveness in Plant Tissue (Food Packaging Applications)

In the context of food packaging, the films must maintain structural integrity, provide an effective barrier against moisture and UV radiation, and, ideally, release bioactive compounds to extend the shelf life of the packaged product. Our results show that AF films, particularly at 10% concentration, exhibited the highest tensile strength (0.326 ± 0.020 MPa) and the greatest UV-blocking capacity (opacity of 0.435 at 600 nm), making them suitable for applications requiring mechanical resistance and photoprotection, such as packaging for light-sensitive products (e.g., oils, dairy products, and beverages). The smooth surface morphology (RMS = 0.192 μm at 10% AF) further suggests good adhesion to hydrophilic surfaces, which is beneficial for coating applications on fruits and vegetables. Conversely, net mass change below 50% “or” net mass gain, which is critical for packaging high-moisture foods, as it prevents structural weakening and maintains barrier properties throughout the product’s shelf life. The enhanced flexibility of 5% AL films (elongation at break = 43.49%) makes them suitable for flexible packaging applications, such as wraps and bags, where adaptability to product shape is required. However, the limited migration of hydrophobic active compounds (bixin, norbixin, and polyphenols) from the hydrophilic starch matrix, as evidenced by the absence of inhibition zones in antimicrobial assays, suggests that the bioactive effects of these films in plant tissue would be primarily contact-dependent rather than diffusional. This contact-active mechanism may be sufficient for reducing surface microbial loads on produce, but further studies are needed to evaluate the release kinetics of active compounds under real packaging conditions, including variations in temperature, relative humidity, and food matrix composition.

### 4.2. Soil-Induced Mass Loss and Disintegration

The soil-induced mass loss tests, while indicative of the films’ susceptibility to microbial and environmental disintegration, should be interpreted with caution. The gravimetric method employed measures the loss of recoverable sample mass, which may include not only possible microbial mineralization (i.e., potential enzymatic cleavage of polymer chains and mineralization to CO_2_ and H_2_O) but also physical disintegration into small fragments that are not recovered during washing, as well as the dissolution of water-soluble components such as glycerol and low-molecular-weight extract residues [44,45]. This methodological limitation is common in studies of starch-based materials and has been acknowledged in previous work [23,35]. Despite this, the observed trends—accelerated mass loss in AF films and retarded mass loss in AL films—provide valuable comparative insights into the relative stability of the formulations under soil conditions. The slower mass loss of AL films is consistent with their denser, more crosslinked structure, which would limit both enzymatic access and physical fragmentation. Future studies should employ complementary techniques such as CO_2_ evolution monitoring (respirometry), molecular weight analysis of residual polymer, and scanning electron microscopy of soil-incubated samples to confirm possible microbial mineralization and distinguish it from physical disintegration. Pure starch films reached complete recoverable mass loss within 21 days, consistent with their inherent susceptibility to enzymatic hydrolysis, but not confirmed by soil microorganisms. The incorporation of AF extracts, particularly at 10% concentration, accelerated mass loss in the initial phase (23.65% mass loss in 7 days), suggesting that the fruit extract acts as a readily accessible carbon source that stimulates microbial activity. In contrast, AL films exhibited slower mass loss kinetics, with a more gradual mass loss over the 28-day period. This behavior is attributed to the formation of hydrogen bonds between the phenolic compounds of the leaf extract and the starch matrix, resulting in a denser, more compact structure that is less accessible to potential enzymatic attack. From a practical standpoint, this tunable mass loss/disintegration behavior offers opportunities for designing films with controlled lifespans: AF-rich formulations may be suitable for short-term applications where rapid disintegration is desired (e.g., single-use food wrappers), while AL-rich formulations could be used in applications requiring longer functional stability (e.g., agricultural mulching films or biomedical devices that require gradual resorption). Notably, the soil pH (6.8 ± 0.2), organic matter content (3.5 ± 0.3%), and sandy loam texture used in this study are representative of agricultural soils in the Peruvian region, providing relevant insights into the environmental fate of these materials under realistic disposal conditions. Importantly, the soil used in this study (pH 6.8 ± 0.2, organic matter 3.5 ± 0.3%, sandy loam texture) is representative of agricultural soils in the Peruvian region, providing realistic insights into the environmental fate of these materials. The native microbial community (bacteria, fungi, and actinomycetes) present in the soil drove the mass loss process, as no external inoculum was added, mimicking real disposal conditions.

### 4.3. Stability and Effectiveness in Culture Media (Antimicrobial Activity)

The antimicrobial assays in culture media revealed a critical limitation of the developed films: the absence of clear inhibition zones against *E. coli*, *S. aureus,* and *C. albicans* indicates that the active hydrophobic compounds (bixin, norbixin, and polyphenols) remain entrapped within the hydrophilic starch matrix rather than diffusing freely into the agar medium. This restricted migration is characteristic of hydrophobic bioactive compounds embedded in hydrophilic polymer networks and underscores the importance of the delivery mechanism. While the contact-inhibitory effect observed at higher extract concentrations (7.5% and 10%) suggests that surface-associated compounds can interact with microorganisms upon direct contact, the effectiveness of these films in liquid culture or in vivo systems would likely be limited without the incorporation of release-enhancing agents or the use of alternative film formulations (e.g., emulsions or multilayers). The higher susceptibility of *S. aureus* (Gram-positive) compared to *E. coli* (Gram-negative) is consistent with the protective role of the outer lipopolysaccharide membrane of Gram-negative bacteria, which acts as a permeability barrier against hydrophobic compounds. This observation highlights the need for strategies to enhance the release of active compounds, such as the incorporation of surfactants, the use of nanoencapsulation, or the surface functionalization of the films to improve their antimicrobial efficacy in complex biological environments.

### 4.4. Structural Mechanisms Governing Antimicrobial Activity

The limited antimicrobial diffusion observed in the *Bixa orellana*-functionalized starch films can be explained by several interconnected structural mechanisms. First, the hydrophilic nature of the starch matrix plays a critical role in restricting the mobility of hydrophobic bioactive compounds. Starch, being a polysaccharide rich in hydroxyl groups, forms an extensive hydrogen-bonding network that creates a polar environment [49]. The hydrophobic carotenoids (bixin and norbixin) and polyphenolic compounds present in the extracts tend to aggregate within the polymer matrix rather than dissolve and diffuse through it, a phenomenon known as hydrophobic entrapment [50]. This effect is analogous to the behavior of essential oils in hydrophilic films, where the hydrophobic droplets remain encapsulated within the continuous polymer phase, resulting in limited release kinetics.

Second, the molecular interactions between the extract components and the starch chains further restrict diffusion. FTIR analysis (Figure 5 and Figure 6) confirmed the formation of hydrogen bonds between the hydroxyl groups of starch and the phenolic –OH groups of the extracts, as evidenced by the shifts in the O–H stretching band (~3400 cm^−1^). These non-covalent interactions effectively anchor the bioactive compounds to the polymer matrix, reducing their molecular mobility and preventing their release into the surrounding medium [51]. Additionally, hydrophobic interactions between the non-polar regions of the carotenoids and the helical structure of amylose may contribute to the formation of inclusion complexes, further immobilizing the active compounds within the starch matrix [52].

Third, the dense and compact structure of the films, particularly at higher extract concentrations, acts as a physical barrier to diffusion. SEM and AFM analyses (Figure 4 and Figure 7) revealed that the incorporation of AL extracts resulted in rougher and more compact surfaces, which may reduce the porosity of the films and hinder the release of active compounds. This structural densification is attributed to the crosslinking effect of phenolic compounds, which can form additional hydrogen bonds and hydrophobic interactions with the starch chains, creating a more tightly packed polymer network [52].

The mechanism of antimicrobial action in these films is therefore predominantly contact-dependent rather than diffusional. When microorganisms come into direct contact with the film surface, the bioactive compounds localized at the interface can interact with the microbial cell wall, disrupting membrane integrity and leading to cell death [53]. This contact-active mechanism is consistent with the visual alterations in film opacity and color observed at higher extract concentrations (7.5% and 10%), particularly against *C. albicans* and *S. aureus*, where direct contact with the film surface appears to inhibit microbial growth.

The differential susceptibility of microorganisms further supports this mechanistic interpretation. The higher susceptibility of *S. aureus* (Gram-positive) compared to *E. coli* (Gram-negative) can be attributed to structural differences in their cell envelopes. Gram-positive bacteria possess a thick peptidoglycan layer that is porous and allows direct access of small molecules to the cytoplasmic membrane. In contrast, Gram-negative bacteria have an additional outer membrane composed of lipopolysaccharides (LPS) and phospholipids, which acts as an effective permeability barrier against hydrophobic compounds [54]. This structural feature limits the interaction of the hydrophobic bioactive compounds with the Gram-negative cell wall, even in a contact-dependent scenario, explaining the lower susceptibility of *E. coli* observed in this study.

In summary, the antimicrobial activity of the *Bixa orellana*-functionalized starch films is governed by a combination of: (1) hydrophobic entrapment of active compounds within the hydrophilic starch matrix, (2) hydrogen bonding and hydrophobic interactions that immobilize the bioactive molecules, (3) structural densification that reduces porosity and limits diffusion, and (4) a contact-dependent mechanism of action that requires direct microbial interaction with the film surface. These structural mechanisms collectively explain the absence of clear inhibition zones in agar diffusion assays and highlight the importance of considering the film’s microstructure when designing antimicrobial biopolymer materials.

### 4.5. Comparison with Existing Antimicrobial Films

The antimicrobial performance of the starch-based films developed in this study can be contextualized by comparing them with other biopolymer films functionalized with natural extracts reported in the literature. For instance, starch-based films incorporated with green tea extract (*Camellia sinensis*) have demonstrated clear inhibition zones against *S. aureus* and *E. coli* due to the high diffusibility of catechins and epigallocatechins, which are relatively hydrophilic polyphenols that can migrate through agar media [55]. Similarly, chitosan-starch films containing oregano essential oil have shown strong antibacterial activity, attributed to the release of volatile terpenoids that diffuse through the agar and create concentration-dependent inhibition zones [56]. In contrast, the limited antimicrobial diffusion observed in our *Bixa orellana*-functionalized films is consistent with reports on starch films incorporating curcumin (from turmeric) or hydrophobic carotenoids, where the active compounds remain predominantly entrapped within the hydrophilic polymer matrix, resulting in contact-dependent rather than diffusional inhibition [57].

When comparing with previous studies on *Bixa orellana* extracts, Venugopalan and Giridhar [16] reported antibacterial activity of annatto seed extracts against *Bacillus subtilis* and *Pseudomonas aeruginosa* using disc diffusion assays, where the extracts were applied directly onto agar without incorporation into a polymer matrix. Similarly, Poma-Castillo et al. [15] demonstrated antifungal activity of ethanolic *Bixa orellana* extracts against *Candida albicans* in solution. However, these studies evaluated the extracts in their free form, where diffusion is not hindered by a polymeric matrix. The entrapment effect observed in our films is a common challenge in the development of active packaging materials, as the polymer matrix acts as a reservoir that controls the release of active compounds. This phenomenon has been extensively documented in starch-based films containing hydrophobic essential oils, where the addition of surfactants or emulsifiers is often required to enhance release and antimicrobial efficacy [58,59].

Despite the absence of clear inhibition zones in agar diffusion assays, the contact-inhibitory effect observed in our films—particularly at higher extract concentrations—is comparable to that reported for starch films containing *Rosmarinus officinalis* (rosemary) extract, which also exhibited limited diffusion but significant surface antimicrobial activity against foodborne pathogens [60]. Furthermore, the higher susceptibility of *S. aureus* (Gram-positive) compared to *E. coli* (Gram-negative) observed in our study is consistent with previous reports on hydrophobic plant extracts, where the outer lipopolysaccharide membrane of Gram-negative bacteria serves as an effective permeability barrier [53,61].

Overall, while the antimicrobial efficacy of our *Bixa orellana*-functionalized films in agar diffusion assays is modest compared to films containing highly diffusible hydrophilic antimicrobials, the films offer significant advantages in terms of UV protection (opacity up to 0.435), mechanical reinforcement (0.326 MPa tensile strength), and moisture resistance (water absorption < 50%), which are not always achieved in films containing highly diffusible antimicrobial agents. This multifunctional performance positions the *Bixa orellana*-functionalized starch films as promising candidates for applications where photoprotection, mechanical strength, and moisture barrier properties are prioritized over diffusional antimicrobial activity, such as in the packaging of dry or low-moisture foods. Future studies should explore strategies to enhance the release of bioactive compounds, including the use of hydrophilic carriers, nanoencapsulation, or the development of multilayer films with a release layer, to broaden their antimicrobial applicability.

### 4.6. Overall Assessment

In summary, the stability and effectiveness of *Bixa orellana*-functionalized starch films are context-dependent and can be tailored by selecting the appropriate plant part (fruit vs. leaf) and concentration. AF films offer superior mechanical strength and UV protection, making them ideal for protective packaging of light-sensitive products, while AL films provide enhanced moisture resistance and flexibility, suitable for flexible packaging applications. The soil disintegration of these films is tunable, with AF formulations promoting faster disintegration and AL formulations extending the material’s environmental lifespan. However, the limited antimicrobial diffusion in culture media highlights a significant challenge for biomedical applications, where the release of active compounds is essential for efficacy. Future work should focus on modifying the starch matrix to improve the release kinetics of bioactive compounds, evaluating the films in real food systems, and conducting ecotoxicity assessments to ensure environmental safety.

### 4.7. Non-Monotonic Mechanical Behavior and Underlying Mechanisms

The strongly non-monotonic mechanical response observed in both AF and AL films (Figure 8 and Figure 9 and Table 3) warrants careful mechanistic interpretation, as it cannot be explained by a simple concentration-dependent reinforcement or plasticization model.

At low concentrations (2.5% *w*/*w*), the addition of both AF and AL extracts led to a notable decrease in tensile strength alongside an increase in elongation at break. This behavior is characteristic of a plasticizing or anti-plasticizing effect, where the small bioactive molecules intercalate between starch chains, weaken inter-chain hydrogen bonding, and increase molecular mobility, thereby reducing stiffness and enhancing ductility. Similar effects have been reported for starch films incorporating low concentrations of phenolic-rich plant extracts [62,63].

At intermediate concentrations (5% *w*/*w*), a recovery in tensile strength was observed, particularly for the AL extract (0.192 MPa), while elongation reached its maximum (43.49%). This suggests that at this specific concentration, the phenolic compounds and flavonoids begin to establish crosslinking hydrogen bonds with the starch matrix, counteracting the plasticizing effect and forming a more cohesive and flexible polymer network [64]. The balance between hydrogen bonding reinforcement and plasticization is optimal at this loading.

However, at 7.5% *w*/*w*, a pronounced deterioration in both tensile strength and elongation was evident for both extracts. This sharp decline can be attributed to molecular overcrowding and the onset of micro-scale phase separation or aggregation of the extract components within the starch matrix. At these higher loadings, the bioactive compounds may self-associate rather than disperse homogeneously, creating stress concentration points and defects that weaken the overall film integrity, reducing both load-bearing capacity and ductility [65]. This phenomenon is commonly observed in polymer composites when filler particles exceed the critical dispersion limit.

Interestingly, at the highest concentration (10% *w*/*w*), the AF and AL extracts diverged in their mechanical behavior. The 10% AF formulation achieved the highest tensile strength among all samples (0.326 MPa), indicating a percolation threshold or a re-organization of the carotenoid-starch network into a reinforcing filler structure at this specific saturation point. The highly hydrophobic, planar structure of bixin and norbixin may allow for strong π-π stacking interactions and hydrophobic associations with the amylose helix, leading to effective stress transfer and reinforcement at high loading [66]. In contrast, the 10% AL film maintained a consistently low tensile strength, suggesting that the highly polar and structurally complex phenolic compounds from the leaf extract reach a solubility limit that disrupts matrix continuity, making the film permanently brittle and weak [67].

These results highlight that the mechanical performance of starch-based films functionalized with plant extracts cannot be assumed to follow a linear progression. Instead, the concentration-dependent balance between plasticization, hydrogen bonding reinforcement, phase separation, and percolation effects determines the final mechanical properties. This underscores the critical importance of optimizing extract loading for specific applications, as excessive or insufficient concentrations may lead to suboptimal performance.

### 4.8. Interpretation of the Net Mass Change Profiles: Coupled Water Uptake and Component Leaching

The decline in net film mass observed after the initial peak in the immersion test (Figure 10) is a critical observation that warrants careful mechanistic interpretation. This behavior cannot be attributed solely to water absorption; rather, it is a clear indication of concurrent leaching of water-soluble components from the film matrix into the aqueous medium.

This phenomenon is well-documented in starch-based films plasticized with glycerol. Glycerol is highly hydrophilic and, upon immersion in water, readily diffuses out of the polymer matrix due to its high solubility and low molecular weight, leading to a net mass loss that can outweigh the mass gained from water absorption [62,65]. Additionally, the alcoholic extracts (AF and AL) contain low-molecular-weight phenolic compounds, flavonoids, and residual sugars that may also be partially soluble in the aqueous medium, further contributing to the observed mass loss [68].

The control film (pure starch) exhibited the most pronounced decline after its peak, consistent with the absence of strong crosslinking interactions that would retain the plasticizer and matrix components. The AF films showed a similar, though less severe, decrease, which may be attributed to the presence of carotenoids—hydrophobic molecules that do not form strong interactions with water and may offer limited retention of the plasticizer.

In marked contrast, the AL films exhibited the lowest net mass values and the least pronounced decrease after the peak. This can be explained by the formation of a denser, hydrogen-bonded network between the starch chains and the phenolic compounds and flavonoids present in the leaf extract. These non-covalent interactions physically entrap the glycerol molecules and low-molecular-weight extract components within the matrix, reducing their mobility and diffusivity into the aqueous medium [69]. Moreover, the crosslinking effect of the phenolics reduces the overall hydrophilicity of the matrix, limiting both water uptake and the dissolution of matrix components.

Therefore, the net mass change curves presented in Figure 10 should not be interpreted as purely “Net mass change kinetics.” Instead, they reflect a competitive dynamic between water absorption and component dissolution. The fact that the AL films maintained stable, low net mass values indicates a superior balance between moisture resistance and structural retention, which is advantageous for packaging applications where exposure to humid or liquid environments is expected.

## 5. Conclusions

This study makes a significant scientific contribution by providing the first systematic comparative analysis of starch-based films functionalized with extracts from two distinct organs of *Bixa orellana*: fruits (AF) and leaves (AL). While previous investigations have explored the antimicrobial and antioxidant properties of *Bixa orellana* extracts in their free form, no prior work has systematically investigated how the biochemical differences between these plant organs influence the structural, morphological, and functional properties of biopolymer films. Our findings demonstrate that the phytochemical divergence between fruits (rich in carotenoids such as bixin and norbixin) and leaves (rich in chlorophyll, phenolics, and flavonoids) translates into distinct and tunable material behaviors, enabling the rational design of films with tailored properties for specific applications.

In conclusion, corn starch-based active films were successfully functionalized with *Bixa orellana* fruit (AF) and leaf (AL) extracts, demonstrating that phytochemical variations between plant organs can be strategically leveraged to tailor the optical, morphological, mechanical, barrier, and bioactive properties of biopolymers. UV-Vis and FTIR analyses confirmed the effective incorporation of bioactive carotenoids (bixin/norbixin), phenolics, and flavonoids that interacted with the starch matrix through hydrogen bonding, imparting strong UV-blocking capability (opacity peaking at 0.435 at 600 nm for 10% AF). Morphologically, fruit extract integrated smoothly to reduce surface roughness (RMS = 0.192 μm at 10% AF) and reinforced the polymeric network to achieve maximum tensile strength (0.326 ± 0.020 MPa), whereas leaf extract induced greater micro-scale surface roughness (RMS = 0.694 μm at 10% AL), enhanced flexibility (43.49 ± 3.5% elongation at break at 5% AL), and significantly improved hydrophobic barrier performance by net mass change below 50% (compared to ∼120% for pure starch). Furthermore, phenolic interactions successfully modulated soil-induced mass loss over 28 days. Although agar diffusion assays revealed no clear inhibition zones against *Escherichia coli*, *Staphylococcus aureus*, or *Candida albicans*—likely due to the entrapment and limited migration of hydrophobic active compounds within the hydrophilic starch matrix—these *Bixa orellana*-functionalized films demonstrate significant potential as sustainable, photoprotective, and moisture-resistant materials for eco-friendly packaging and biomedical applications. Compared to existing antimicrobial films based on highly diffusible hydrophilic agents (e.g., green tea extract, chitosan), our *Bixa orellana*-functionalized films exhibit modest antimicrobial diffusion but offer superior multifunctional performance, including high UV-blocking capacity, mechanical reinforcement, and moisture resistance. This makes them particularly suitable for applications where surface contact inhibition and photoprotection are prioritized over diffusional antimicrobial activity, such as in packaging for dry or light-sensitive products. While the soil mass loss data provide useful comparative information on the environmental stability of the films, the results should be interpreted as indicators of disintegration rather than definitive biodegradation/mineralization. Future work should incorporate respirometric measurements to confirm mineralization rates and assess the ecological impact of the disintegration products.

## Figures and Tables

**Figure 1:**
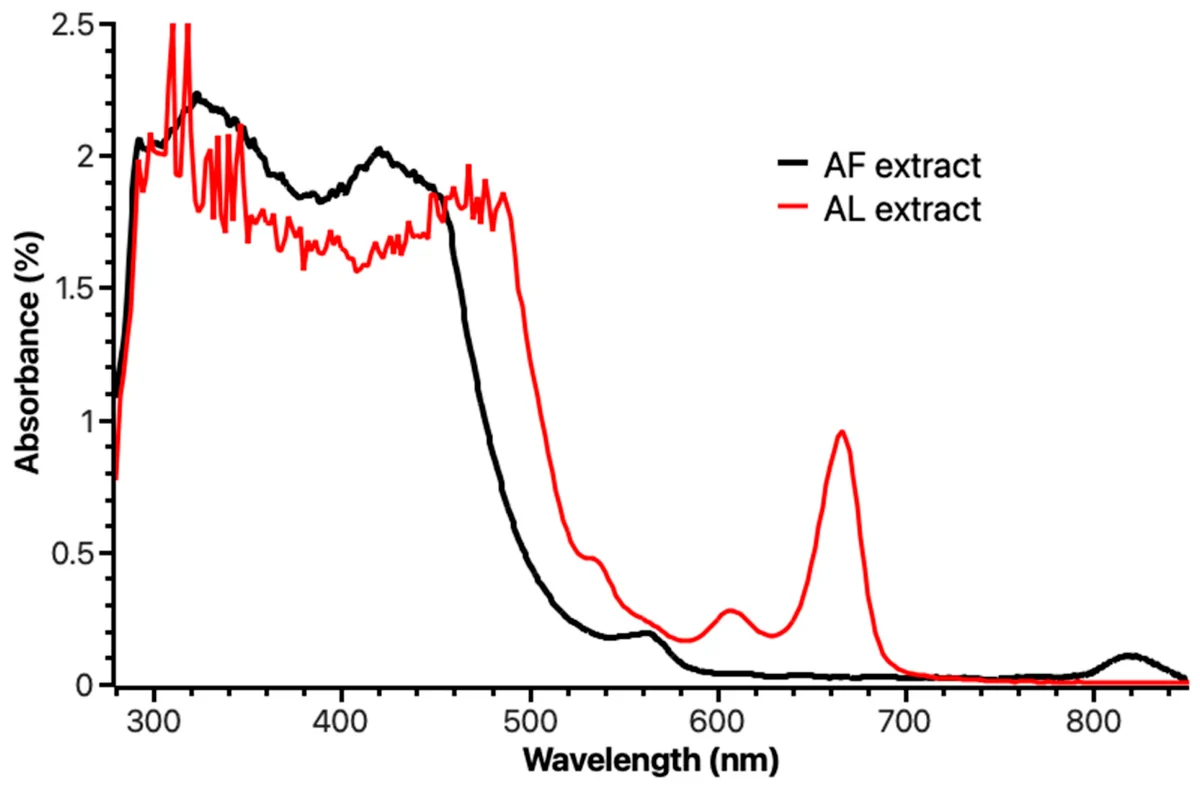
UV-Vis absorbance of alcoholic extracts of AF (black) and AL (red).

**Figure 2:**
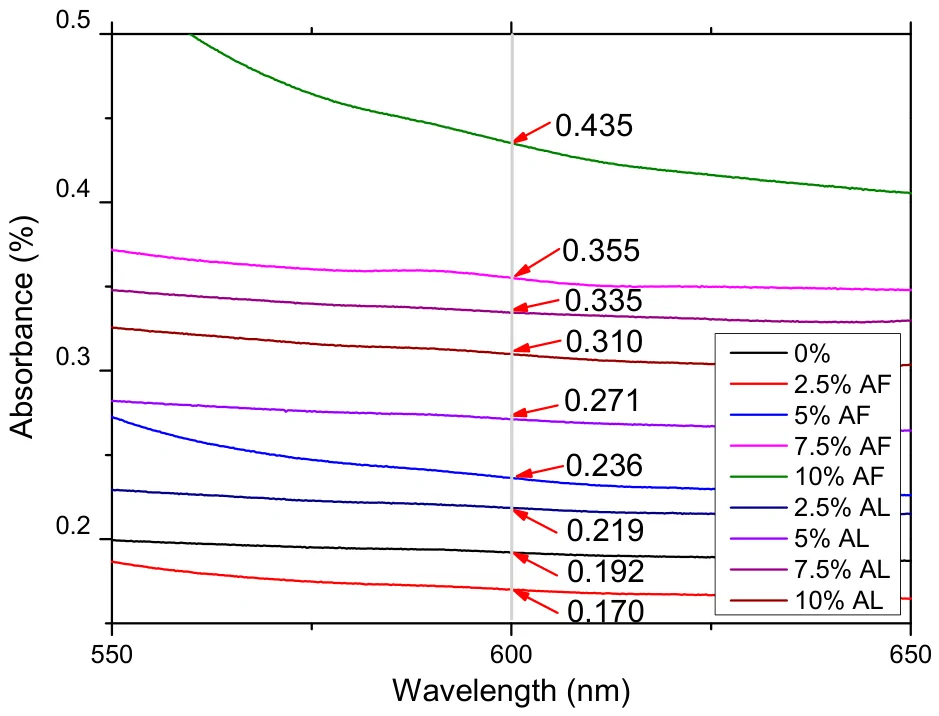
UV-Vis Absorbance of films of corn starch (0%), 2.5% AF, 5% AF, 7.5% AF, 10% AF and 2.5% AL, 5% AL, 7.5% AL, 10% AL.

**Figure 3:**
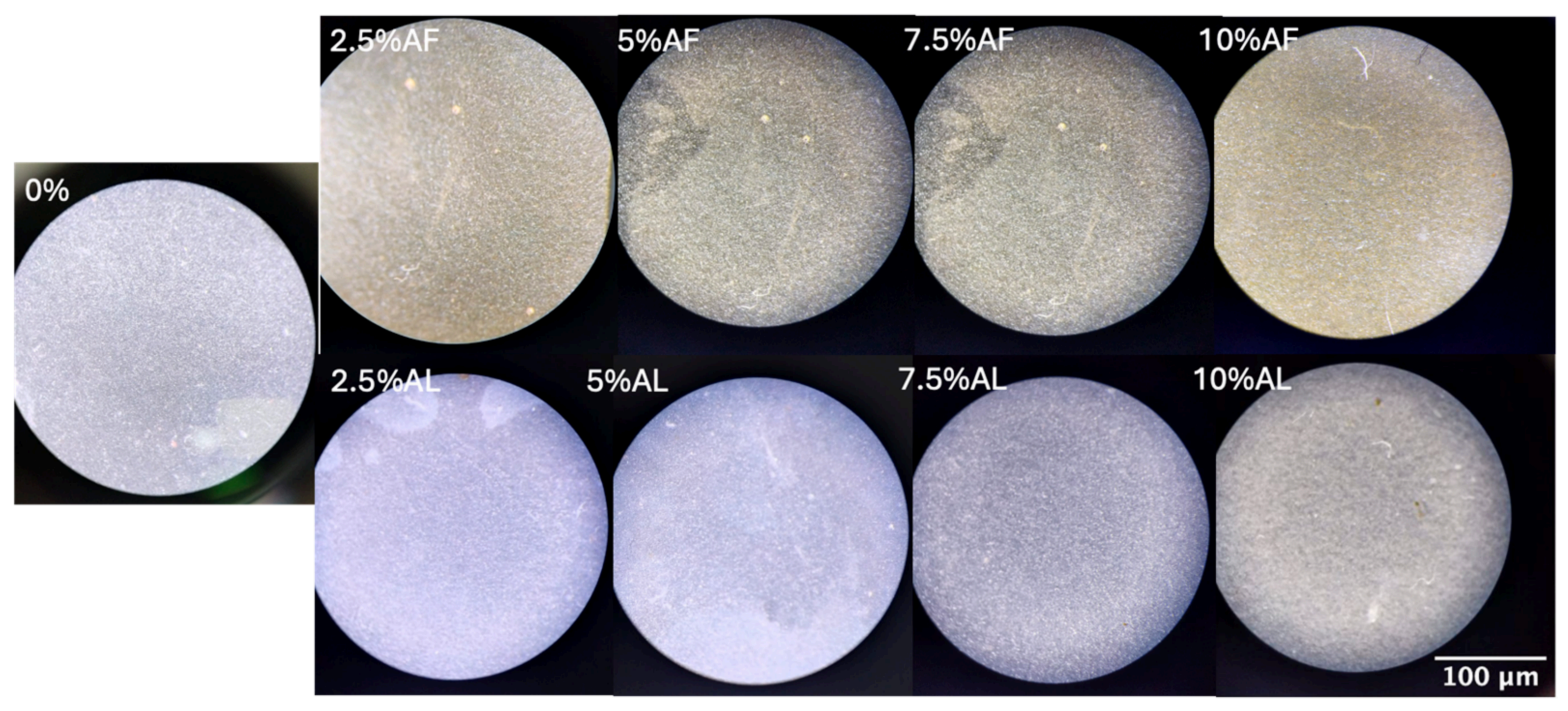
Microscope images of films of corn starch (0%), 2.5% AF, 5% AF, 7.5% AF, 10% AF and 2.5% AL, 5% AL, 7.5% AL, 10% AL. Magnification: 40×. Scale bar: 100 µm.

**Figure 4:**
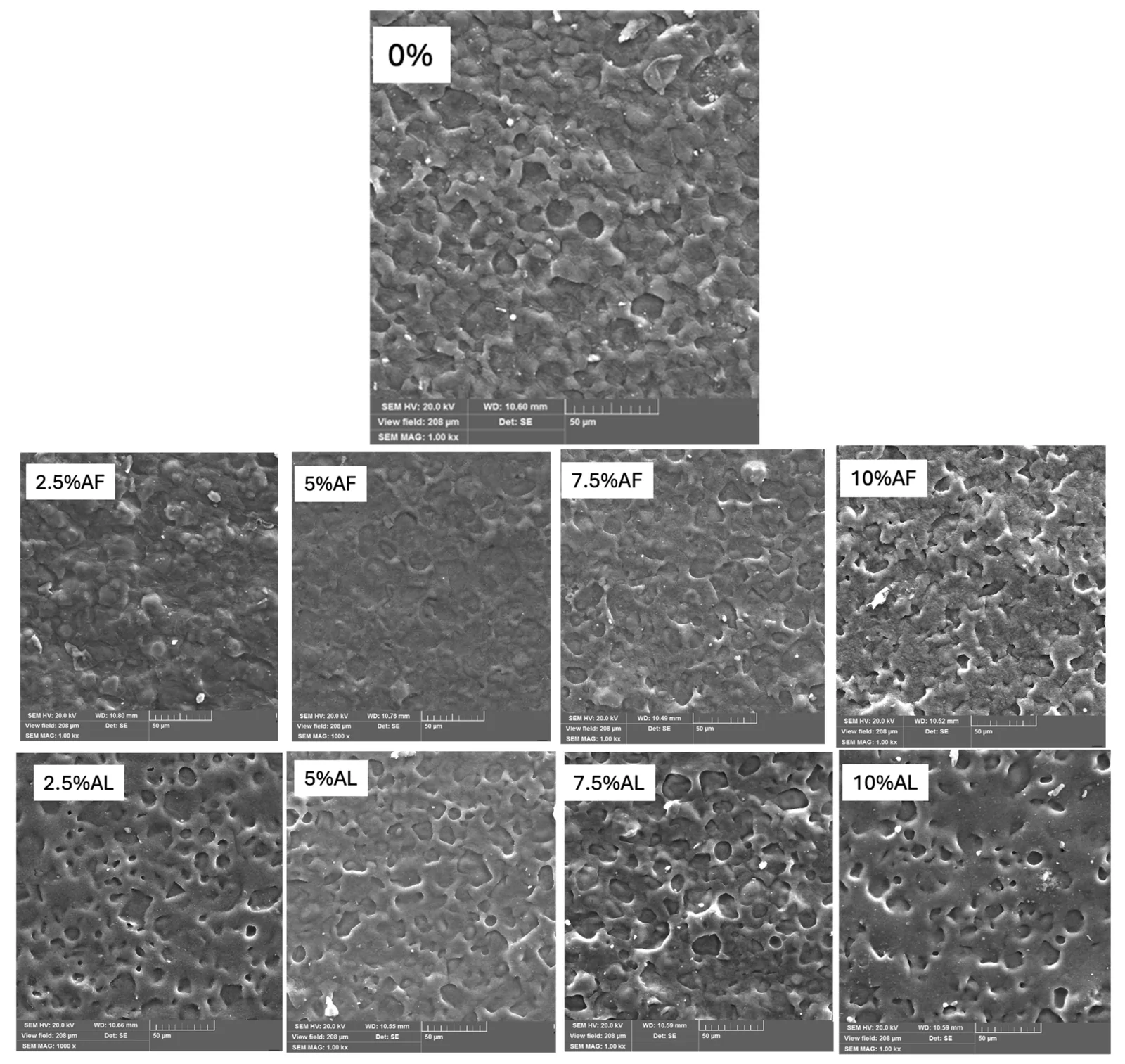
Morphological images of the samples: corn starch (0%), 2.5% AF, 5% AF, 7.5% AF, 10% AF and 2.5% AL, 5% AL, 7.5% AL, 10% AL at a magnification of 1kx. Scale bar: 50 µm.

**Figure 5:**
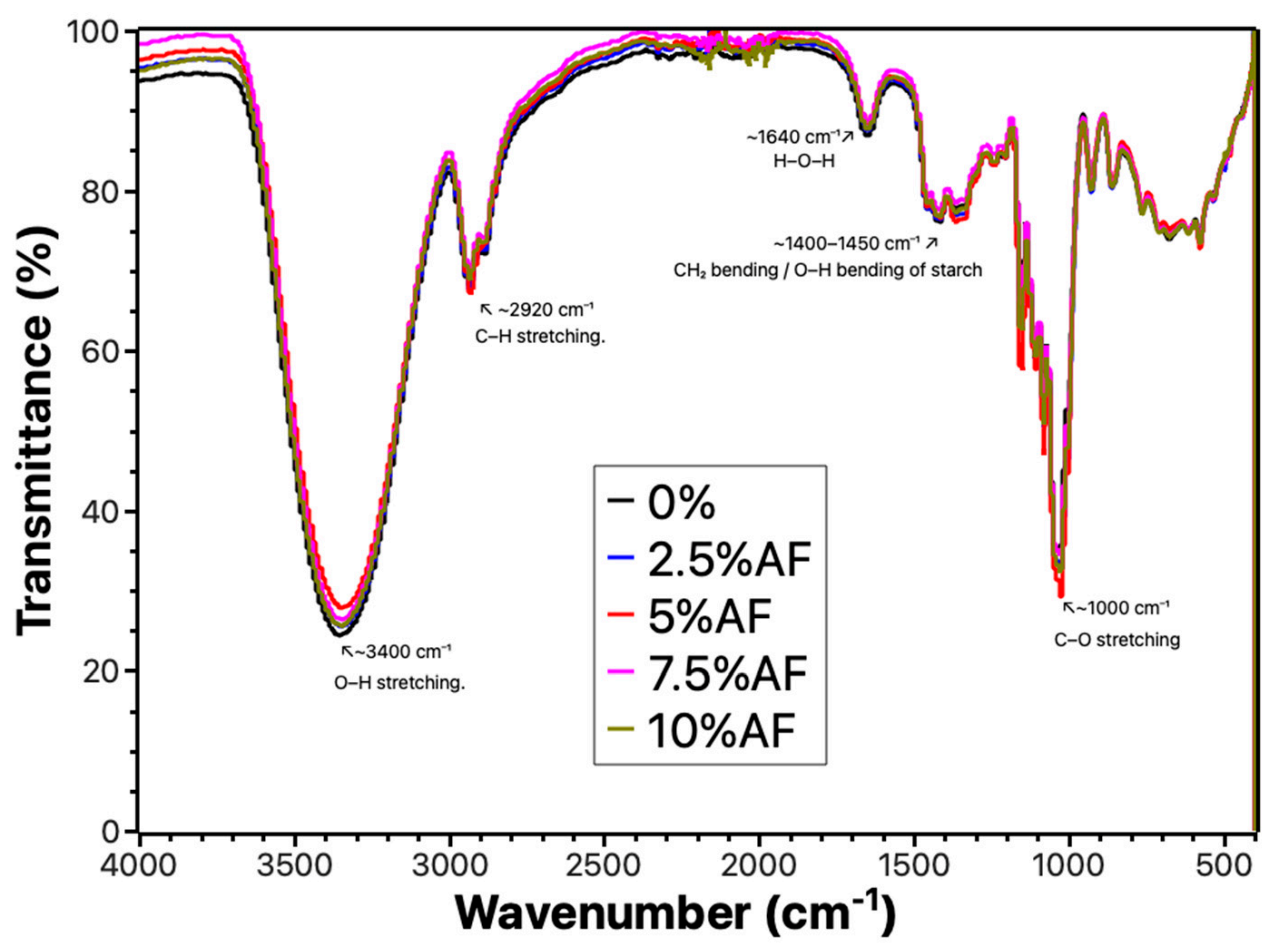
FTIR spectra of corn starch films: control (0%) and films with different concentrations (2.5%, 5%, 7.5%, and 10%) of the AF extract. Characteristic bands: O–H stretching (~3400 cm^−1^), C–H stretching (~2920 cm^−1^), H–O–H bending of bound water (~1640 cm^−1^; possible minor overlap with C=O/C=C of phenolic compounds), CH_2_/O–H bending (~1400–1450 cm^−1^), and C–O stretching (~1000 cm^−1^). Subtle changes in position and intensity indicate hydrogen bonding interactions between the starch and the bioactive compounds.

**Figure 6:**
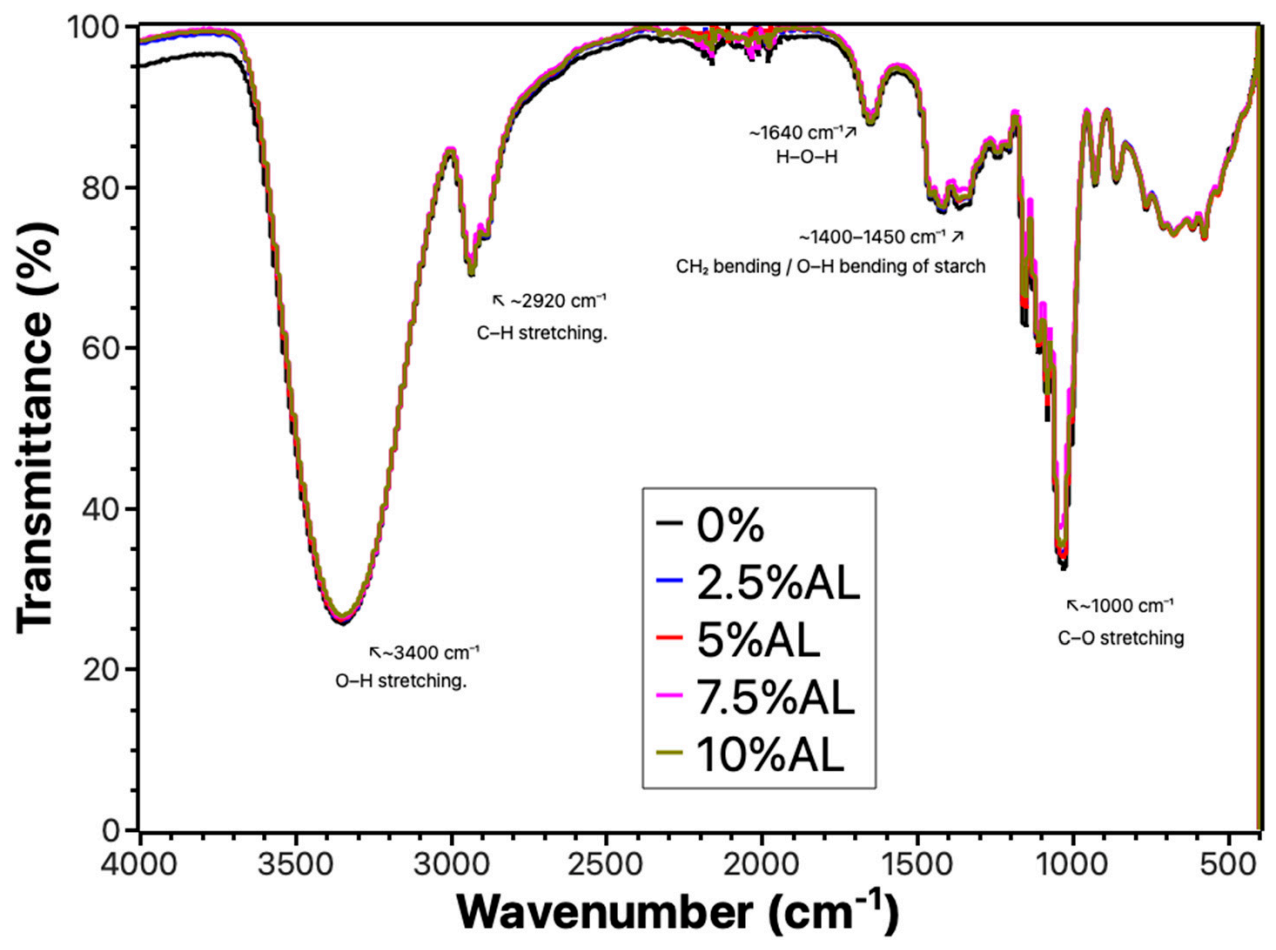
FTIR spectra of corn starch films: control (0%) and films with different concentrations (2.5%, 5%, 7.5% and 10%) of the AL extract. The band assignments are the same as in Figure 5. Changes are observed in the O–H and C–O region consistent with non-covalent interactions between the starch and the phenolic/flavonoid compounds of the leaf extract.

**Figure 7:**
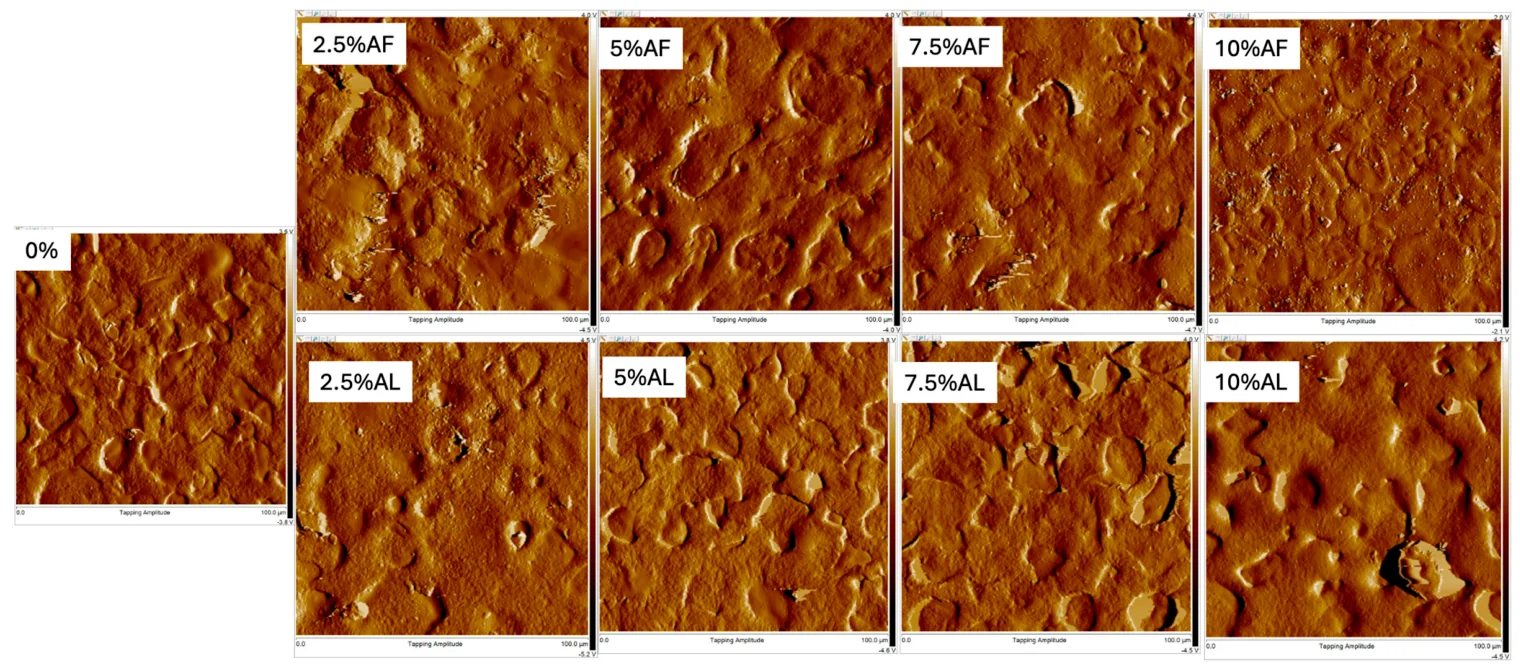
Atomic force microscopy (AFM) of 5 μm × 5 μm sections of the samples: corn starch (0%), 2.5% AF, 5% AF, 7.5% AF, 10% AF and 2.5% AL, 5% AL, 7.5% AL, 10% AL.

**Figure 8:**
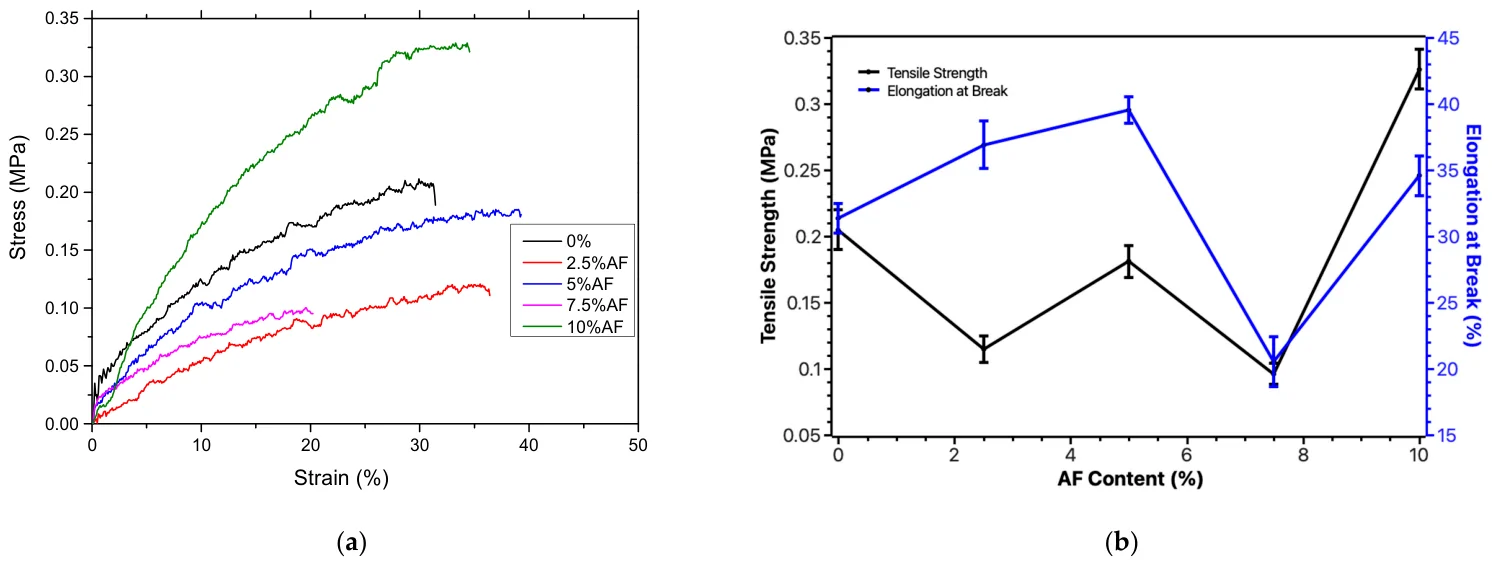
(**a**) Stress–strain curves and (**b**) tensile curve of the films: corn starch (0%), 2.5% AF, 5% AF, 7.5% AF, 10% AF.

**Figure 9:**
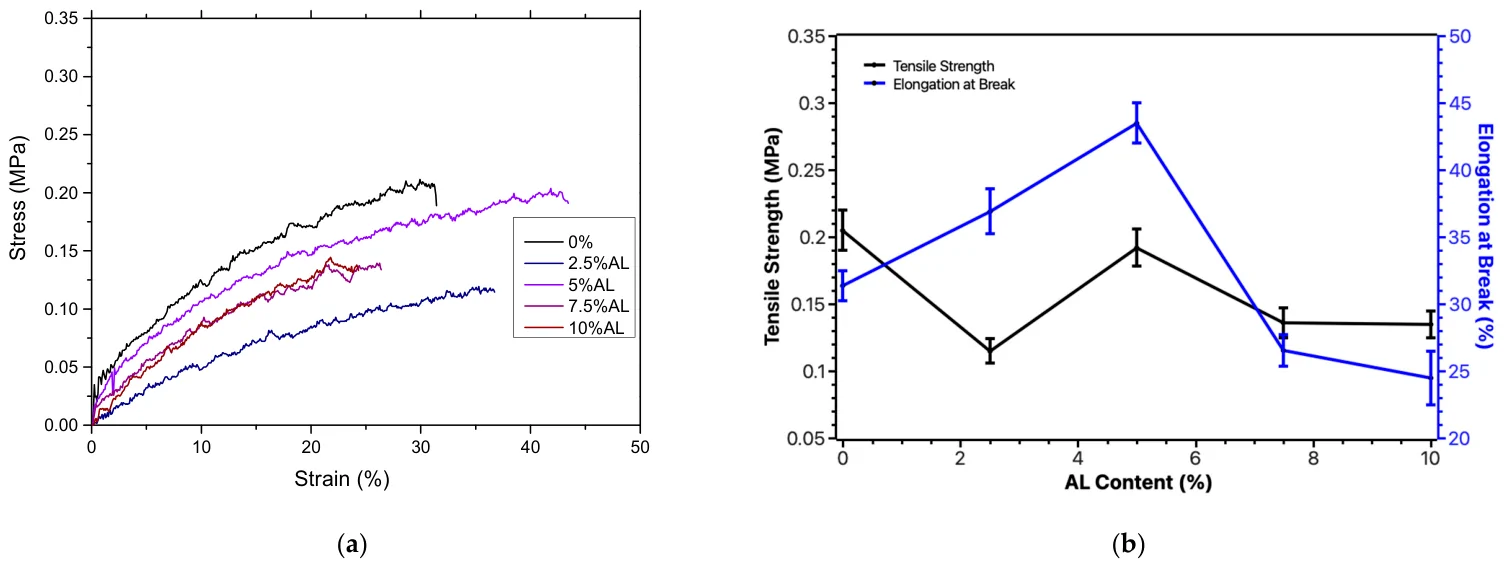
(**a**) Stress–strain curves and (**b**) tensile curve of the films: corn starch (0%), 2.5% AL, 5% AL, 7.5% AL, 10% AL.

**Figure 10:**
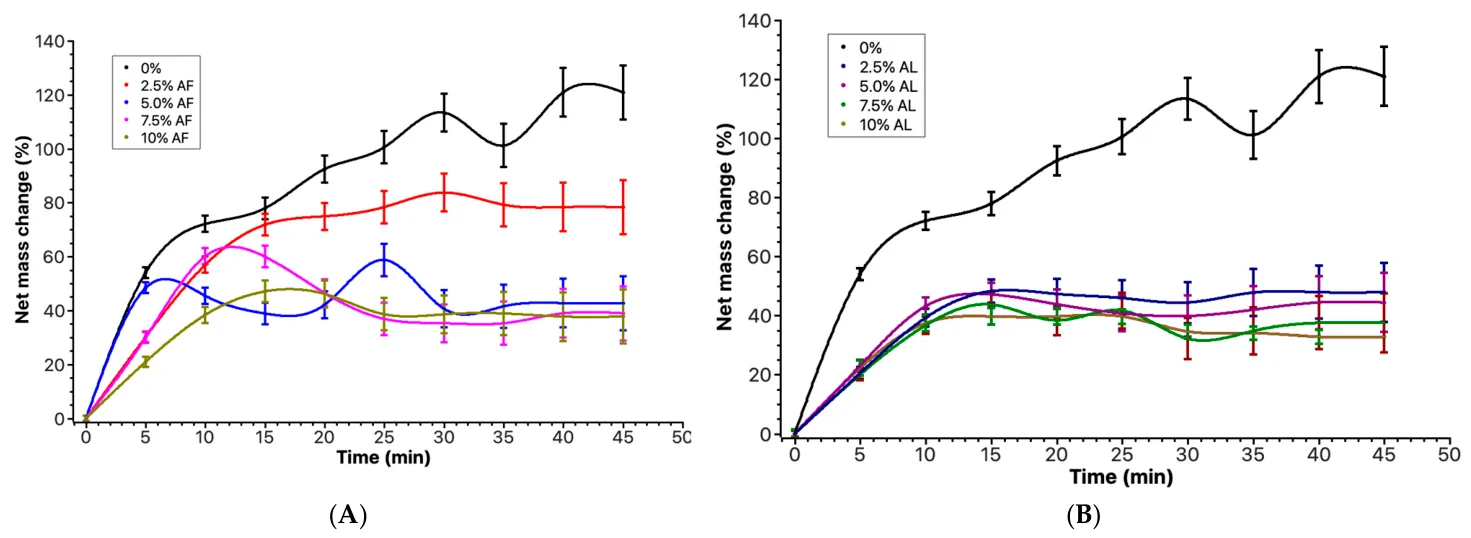
Net mass change kinetics percent curves of the films: corn starch (0%), (**A**) 2.5% AF, 5% AF, 7.5% AF, 10% AF and (**B**) 2.5% AL, 5% AL, 7.5% AL, 10% AL. Data are presented as mean ± standard deviation (SD) from three independent replicates (n = 3). Error bars represent the standard deviation at each time point.

**Figure 11:**
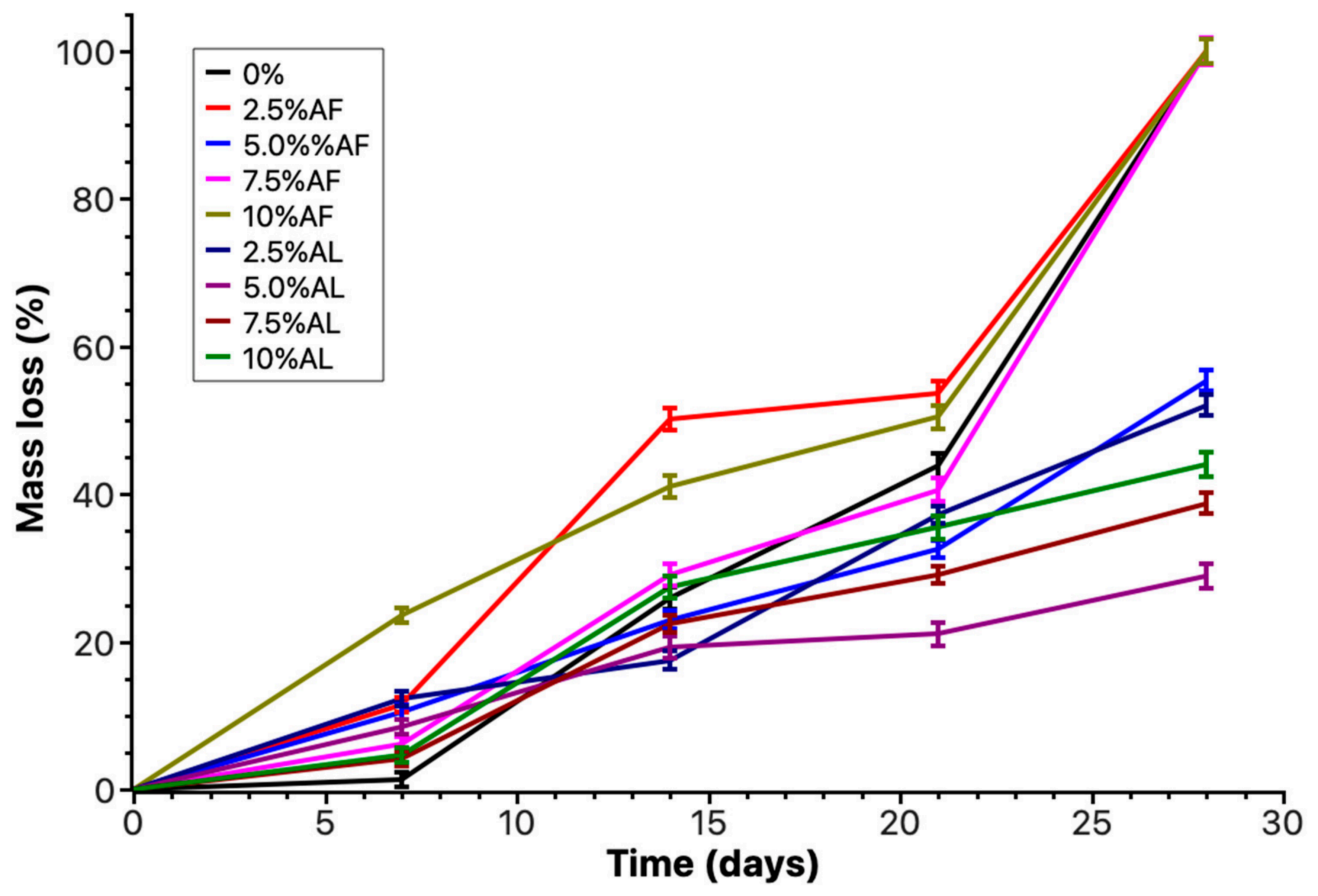
Soil-induced mass loss (disintegration) of corn starch films: control (0%), 2.5% AF, 5% AF, 7.5% AF, 10% AF, and 2.5% AL, 5% AL, 7.5% AL, 10% AL, over 28 days of soil burial. Data are presented as mean ± standard deviation (SD) from three independent replicates (n = 3). Error bars represent the standard deviation at each time point. The gravimetric method measures the loss of recoverable mass, which may include disintegration, physical fragmentation, and dissolution; therefore, results are reported as mass loss/disintegration, not as definitive biodegradation/mineralization.

**Figure 12:**
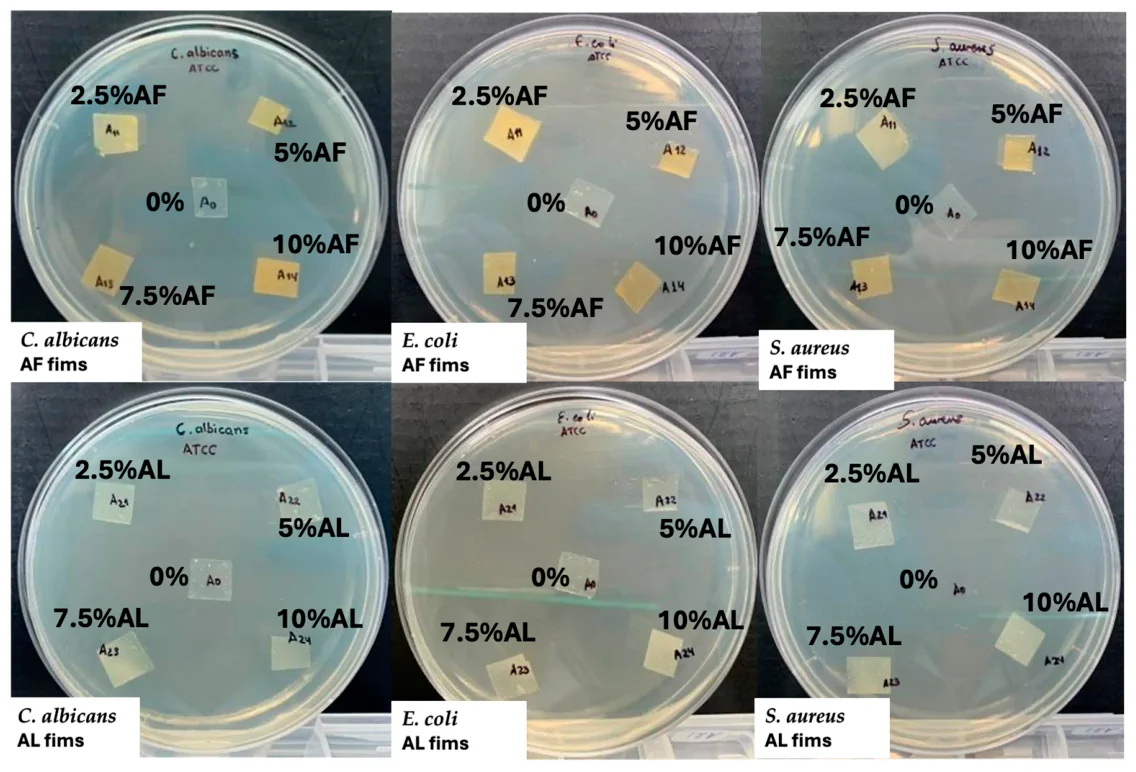
Evaluation of the antibacterial activity against *Candida albicans* of control corn starch films (0%) and films incorporated with different concentrations (2.5%, 5%, 7.5%, and 10%) of AF and AL.

**Table 1:** Composition ratios of films of corn starch 0%, corn starch with AF extract 2.5%, 5%, 7.5%, 10% and with AL extract 2.5%, 5%, 7.5%, 10%.

Sample	
0%	Starch
2.5% AF	Starch + 2.5% *Bixa orellana* fruit extract
5% AF	Starch + 5% *Bixa orellana* fruit extract
7.5% AF	Starch + 7.5% *Bixa orellana* fruit extract
10% AF	Starch + 10% *Bixa orellana* fruit extract
2.5% AL	Starch + 2.5% *Bixa orellana* leaf extract
5% AL	Starch + 5% *Bixa orellana* leaf extract
7.5% AL	Starch + 7.5% *Bixa orellana* leaf extract
10% AL	Starch + 10% *Bixa orellana* leaf extract

**Table 2:** Comparison of RMS of films of corn starch (0%), 2.5% AF, 5% AF, 7.5% AF, 10% AF and 2.5% AL, 5% AL, 7.5% AL, 10% AL.

Sample	RMS (μm)
0%	0.590
2.5% AF	0.571
5% AF	0.553
7.5% AF	0.482
10% AF	0.192
2.5% AL	0.530
5% AL	0.624
7.5% AL	0.592
10% AL	0.694

**Table 3:** Tensile test results on materials of films of corn starch (0%), 2.5% AF, 5% AF, 7.5% AF, 10% AF and 2.5% AL, 5% AL, 7.5% AL, 10% AL.

Sample	Tensile Strength (MPa)	Strain (%)	Statistical Group	Comment
0%	0.205 ± 0.015	31.4 ± 2.1	^b^	Control, moderate resistance
2.5%AF	0.115 ± 0.010	36.9 ± 2.8	^c^	Lower resistance, greater deformation
5%AF	0.181 ± 0.012	39.5 ± 3.0	^b,c^	Slight improvement in resistance, lower ductility
7.5%AF	0.096 ± 0.008	20.5 ± 1.9	^d^	Low resistance, stable behavior
10%AF	0.326 ± 0.020	34.6 ± 2.5	^a^	Greater resistance, good deformation
2.5%AL	0.115 ± 0.009	36.9 ± 2.7	^c^	High deformation, reduced resistance
5%AL	0.192 ± 0.014	43.5 ± 3.5	^b^	Behavior similar to control
7.5%AL	0.136 ± 0.011	26.5 ± 2.2	^c,d^	Low resistance, lower ductility
10%AL	0.135 ± 0.010	24.5 ± 2.0	^c,d^	Overall decrease in mechanical properties

The letter “^a^” is assigned to the group with the highest value (10% AF = 0.326 MPa). Subsequent letters (^b^, ^c^, ^d^) are assigned to groups with significantly different values according to Tukey’s post hoc test.

**Table 4:** Comparison of thickness, density of films of corn starch (0%), 2.5% AF, 5% AF, 7.5% AF, 10% AF and 2.5% AL, 5% AL, 7.5% AL, 10% AL.

Sample	Mass (g)	Width (mm)	Length (mm)	Thickness (mm)	Volume (mm^3^)	Density (g/cm^3^)
0%	0.0866 ± 0.0001	19.78 ± 0.001	20.09 ± 0.001	0.17 ± 0.001	67.554634	1.282
2.5% AF	0.0913 ± 0.0001	19.6 ± 0.001	19.3 ± 0.001	0.21 ± 0.001	79.4388	1.149
5% AF	0.0491 ± 0.0001	20.05 ± 0.001	19.41 ± 0.001	0.1 ± 0.001	38.91705	1.262
7.5% AF	0.0842 ± 0.0001	18.39 ± 0.001	18.02 ± 0.001	0.18 ± 0.001	59.649804	1.412
10% AF	0.0981 ± 0.0001	21.17 ± 0.001	19.49 ± 0.001	0.18 ± 0.001	74.268594	1.321
2.5% AL	0.0965 ± 0.0001	20.19 ± 0.001	19.87 ± 0.001	0.18 ± 0.001	72.211554	1.336
5% AL	0.0994 ± 0.0001	20.12 ± 0.001	21.72 ± 0.001	0.2 ± 0.001	87.40128	1.137
7.5% AL	0.0861 ± 0.0001	19.5 ± 0.001	19.47 ± 0.001	0.16 ± 0.001	60.7464	1.417
10% AL	0.0868 ± 0.0001	18.58 ± 0.001	20.25 ± 0.001	0.17 ± 0.001	63.96165	1.357

Note: 1 g/mm^3^ = 1000 g/cm^3^.

## Data Availability

The original contributions presented in this study are included in the article. Further inquiries can be directed to the corresponding author.

## Abbreviations

The following abbreviations are used in this manuscript:

FTIR	Fourier Transform Infrared Spectroscopy
SEM	Scanning Electron Microscopy
UV-Vis	Ultraviolet–Visible spectrophotometry
MPa	MegaPascals
